# Elucidation of an anaerobic pathway for metabolism of l-carnitine–derived γ-butyrobetaine to trimethylamine in human gut bacteria

**DOI:** 10.1073/pnas.2101498118

**Published:** 2021-08-06

**Authors:** Lauren J. Rajakovich, Beverly Fu, Maud Bollenbach, Emily P. Balskus

**Affiliations:** ^a^Department of Chemistry and Chemical Biology, Harvard University, Cambridge, MA 02138

**Keywords:** microbiota, trimethylamine, l-carnitine

## Abstract

Trimethylamine (TMA) is an important gut microbial metabolite strongly associated with human disease. There are prominent gaps in our understanding of how TMA is produced from the essential dietary nutrient l-carnitine, particularly in the anoxic environment of the human gut where oxygen-dependent l-carnitine–metabolizing enzymes are likely inactive. Here, we elucidate the chemical and genetic basis for anaerobic TMA generation from the l-carnitine–derived metabolite γ-butyrobetaine (γbb) by the human gut bacterium *Emergencia timonensis*. We identify a set of genes up-regulated by γbb and demonstrate that the enzymes encoded by the induced γbb utilization (*bbu*) gene cluster convert γbb to TMA. The key TMA-generating step is catalyzed by a previously unknown type of TMA-lyase enzyme that utilizes a putative flavin cofactor to catalyze a redox-neutral transformation. We identify additional cultured and uncultured host-associated bacteria that possess the *bbu* gene cluster, providing insights into the distribution of anaerobic γbb metabolism. Lastly, we present genetic, transcriptional, and metabolomic evidence that confirms the relevance of this metabolic pathway in the human gut microbiota. These analyses indicate that the anaerobic pathway is a more substantial contributor to TMA generation from l-carnitine in the human gut than the previously proposed aerobic pathway. The discovery and characterization of the *bbu* pathway provides the critical missing link in anaerobic metabolism of l-carnitine to TMA, enabling investigation into the connection between this microbial function and human disease.

## Significance

Trimethylamine (TMA) is a disease-associated metabolite produced in the human body exclusively by microbes. Gut microbes generate TMA from essential nutrients consumed in the human diet, including l-carnitine. However, our understanding of the biochemical mechanisms involved in these transformations is incomplete. In this work, we define the biochemical pathway and genetic components in gut bacteria required for anaerobic production of TMA from γ-butyrobetaine, a metabolite derived from l-carnitine. This discovery identifies a new type of TMA-producing enzyme and fills a critical gap in our knowledge of l-carnitine metabolism to TMA in the anaerobic environment of the human gut. This knowledge will enable evaluation of the link between l-carnitine metabolism and human disease and the design of potential therapeutics.

The human gut microbiota collectively synthesizes an array of small molecule metabolites. The metabolic output of this microbial community varies substantially between individual human subjects, and specific metabolites are strongly associated with health and disease (1–3). In many cases, however, we lack both a molecular understanding of how gut microbial metabolites influence human physiology and how the metabolites themselves are produced. These gaps in knowledge limit our ability to establish causative effects of microbial metabolites in human disease and to develop microbiota-based strategies to improve human health. Identification of the specific organisms, genes, and enzymes responsible for metabolite production is needed to accurately profile specific metabolic functions in microbial communities, to experimentally investigate links to disease, and to modulate the metabolic output of the gut microbiota.

**Fig. 1.**
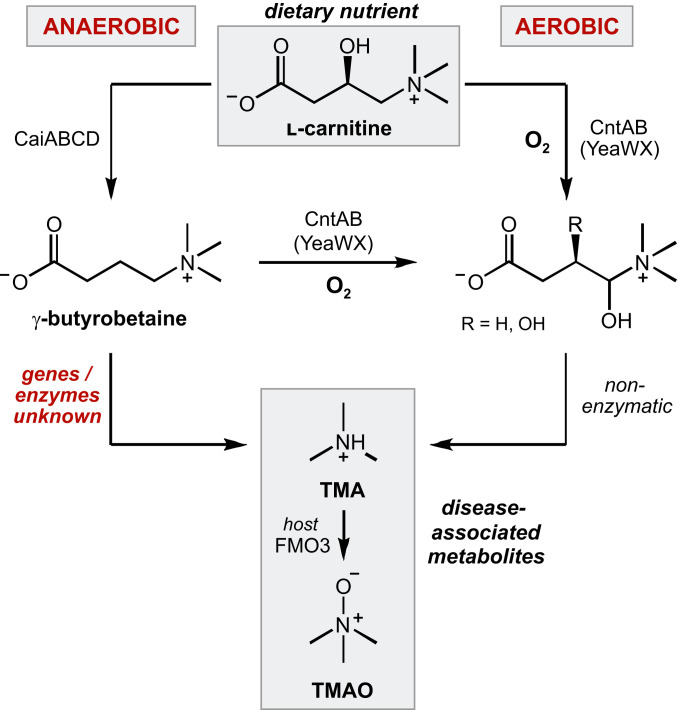
Anaerobic and aerobic bacterial metabolic pathways producing TMA from l-carnitine.

Trimethylamine (TMA) is a gut microbial metabolite that has been strongly associated with human disease. It is derived from gut microbial transformations of dietary nutrients including phosphatidylcholine, choline, l-carnitine, betaine, and TMA *N*-oxide (TMAO) (4–9). Microbially produced TMA is absorbed by the host in the gastrointestinal tract, enters hepatic circulation, and is oxidized to TMAO in the liver by the flavin-dependent monooxygenase FMO3 (10). Genetic mutations in the human *FMO3* gene lead to accumulation of TMA in the body, causing the metabolic disorder trimethylaminuria or fish malodor syndrome (11). In addition, elevated plasma levels of TMA and TMAO have been associated with multiple human diseases, including cardiovascular, chronic kidney, and nonalcoholic fatty liver diseases, obesity, type II diabetes, and colorectal cancer (12). Especially strong correlations between TMAO and its precursors have been demonstrated for cardiovascular disease (CVD). Elevated plasma levels of dietary TMA precursors were also associated with disease risk, but only when they co-occurred with elevated TMAO levels (6, 7, 9). Furthermore, oral administration of the TMA precursors phosphatidylcholine, choline, and carnitine to atherosclerosis-prone mice resulted in development of atherosclerotic plaques in a gut microbiota-dependent fashion (6, 7). Direct oral administration of TMAO in these mice similarly resulted in phenotypes of atherosclerosis (6). These observations suggest a causal role for TMAO in animal models of CVD and that gut microbial metabolism is a crucial factor contributing to pathogenesis. However, a causative role of TMA or TMAO in the development or exacerbation of complex diseases has not yet been definitively established in humans. Deciphering the contribution of TMA production to human disease clearly necessitates a better understanding of the gut microbial metabolic pathways that generate this small molecule.

l-Carnitine is an important dietary precursor to TMA, and its metabolism by gut microbes is associated with CVD (13). An essential nutrient for the host, l-carnitine plays a key role in fatty acid β-oxidation by transporting fatty acids across the mitochondrial membrane for metabolism (5, 14). Although it is produced endogenously, humans must uptake additional l-carnitine in the diet to support cellular function (5, 14). The major sources of l-carnitine are animal-based products, especially red meat (15), but it is also ingested as a supplement for enhanced physical performance (16, 17). In contrast to the host which cannot breakdown l-carnitine, gut bacteria metabolize this molecule in multiple ways. The most well-known conversion of l-carnitine involves the production of TMA; however, the human gut bacterium *Eubacterium limosum* was also recently reported to demethylate l-carnitine (18). Studies in rats and human subjects demonstrated that a large proportion of dietary l-carnitine is converted to TMA and that this metabolism is dependent on the gut microbiota (7, 19, 20). In addition, these studies noted accumulation of an intermediate metabolite identified as γ-butyrobetaine (γbb) that was produced by the gut microbiota (19–21). Furthermore, γbb was shown to be a proatherogenic metabolite in mouse models like its precursor l-carnitine (21). The well-characterized metabolic pathway that converts l-carnitine to γbb is encoded by the *cai* gene operon (Fig. 1) and is used during anaerobic respiration by facultative anaerobic Proteobacteria such as *Escherichia coli*, *Salmonella typhimurium*, and *Proteus mirabilis* (5). The microbial genes and enzymes that are responsible for generating TMA from l-carnitine–derived γbb, however, are not fully elucidated.

Specifically, there is a significant gap in our understanding of the molecular basis for TMA production from l-carnitine and γbb precursors under anaerobic conditions. Select facultative anaerobic Proteobacteria and Actinobacteria possess an iron-dependent Rieske-type monooxygenase (CntA) that can directly convert l-carnitine to TMA (21–23). This enzyme uses dioxygen to hydroxylate l-carnitine at the C4 position, followed by nonenzymatic formation of an aldehyde through elimination of TMA (Fig. 1). Whereas l-carnitine was the only substrate tested for activity with CntA from *Acinetobacter* *baumannii* (22), the *E. coli* homolog, also known as YeaW (71% amino acid identity), was shown to produce low levels of TMA from both l-carnitine and γbb (21). Although CntA activity was originally proposed to represent the major mechanism for conversion of l-carnitine to TMA by the human gut microbiota, this conclusion has been called into question. This enzyme strictly requires dioxygen for catalysis; however, dioxygen levels in the colon lumen are <1 mm Hg (24). In addition, a study in humans showed that plasma TMAO levels after a carnitine challenge were not correlated with *cntA* gene abundance in gut microbiomes (25). Notably, TMA production from l-carnitine and γbb was demonstrated in anaerobic ex vivo incubations of mice cecal and colon tissues (21). Finally, the human gut isolate *Emergencia timonensis*, an obligate anaerobe that does not encode a CntA homolog, was recently found to metabolize γbb to TMA under strictly anaerobic growth conditions (13). Collectively, this information indicates the existence of an as-yet-uncharacterized anaerobic pathway for γbb metabolism in the human gut microbiota.

We present here the identification and characterization of the metabolic pathway, genes, and enzymes responsible for anaerobic TMA production from γbb in *E. timonensis*. The enzyme catalyzing the key C–N bond cleavage reaction that generates TMA is a flavin-dependent, acyl-coenzyme A (CoA) dehydrogenase-like enzyme that uses the activated CoA thioester of γbb as its substrate. This chemically challenging reaction generates the intermediate crotonyl-CoA, which is further metabolized by *E. timonensis* for anaerobic respiration and as a source of carbon and energy. Homologous gene clusters for γbb metabolism are present in other cultured and uncultured host-associated bacteria from the Clostridiales order. We find that anaerobic γbb metabolism is prevalent in human gut microbiomes and is likely a major underappreciated contributor of l-carnitine–derived TMA. Together, this work expands our knowledge of TMA-producing enzymes, pathways, and organisms, providing a more complete understanding of microbial TMA production in the anoxic human gut. These findings identify potential targets for manipulation of this microbial function and will help resolve the major dietary and microbial contributors to TMA production.

## Results

### γbb Induces Expression of a Candidate Gene Cluster in *E. timonensis* SN18.

**Fig. 2.**
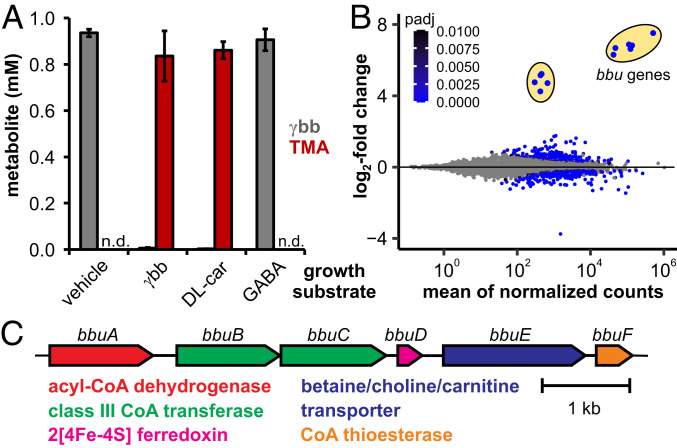
γbb induces expression of the *bbu* gene cluster in *E. timonensis* SN18. (*A*) Bar plots of γbb and TMA concentrations after a 3-h incubation of 1 mM γbb in resting cell suspensions of *E. timonensis* SN18 previously cultured in media supplemented with 1 mM γbb, dl-carnitine, GABA, or 1× PBS (vehicle). Error bars represent the SD from the mean of three biological replicates. (*B*) Differential gene expression from *E. timonesis* SN18 cultures supplemented with γbb or treated with a vehicle at an OD = 0.7, plotted against the mean of normalized counts from three biological replicates. The two sets of differentially up-regulated genes are circled in yellow. Genes with an adjusted *P* > 0.01 (Wald test) comparing γbb- and vehicle-induced cells are represented by gray circles. (*C*) The *bbu* gene cluster and automated protein function annotations.

Only a single cultured bacterium, a human fecal isolate of *E. timonensis*, has been reported to produce TMA from γbb under anaerobic conditions (13). We began our efforts to identify the genes and enzymes responsible for γbb metabolism by testing the type strain *E. timonensis* SN18 for this activity. Indeed, anaerobic cultures of *E. timonensis* SN18 supplemented with γbb demonstrated complete consumption of this substrate and production of a stoichiometric amount of TMA (*SI Appendix*, Fig. S1). When the cultures were supplemented with [*N*-(CD_3_)_3_]-γbb, deuterium-labeled TMA was detected by liquid chromatography–tandem mass spectrometry (LC–MS/MS) (*SI Appendix*, Fig. S1), confirming its origin from γbb. Resting cell suspensions of *E. timonensis* SN18 also fully converted γbb to TMA (Fig. 2*A*). However, this activity was only observed when the cells had been cultured in medium containing γbb; resting cell suspensions of *E. timonensis* SN18 cultured in the absence of γbb were unable to consume γbb and did not generate TMA (Fig. 2*A*). Together, these results suggest that γbb induces expression of the genes involved in its metabolism.

We also evaluated the ability of *E. timonensis* SN18 to metabolize dl-carnitine and γ-aminobutyric acid (GABA), close structural homologs of γbb. Neither of these substrates was consumed during growth (*SI Appendix*, Fig. S1) nor in resting cell suspensions of cultures supplemented with γbb (*SI Appendix*, Fig. S1). Next, we tested whether these substrate analogs could induce expression of the γbb metabolic enzymes. Resting cell suspensions of cultures supplemented with GABA did not exhibit γbb metabolism; however, resting cell suspensions from cultures supplemented with dl-carnitine did convert γbb to TMA (Fig. 2*A*). Thus, while dl-carnitine appears to induce expression of the genes responsible for γbb metabolism in *E. timonensis*, the metabolic enzymes are unable to use dl-carnitine as a substrate for TMA generation.

The inducible metabolism of γbb in *E. timonensis* SN18 suggested that the genes involved in this pathway could be discovered using RNA-sequencing (RNA-seq). Only two sets of genes were both highly and differentially expressed in γbb-induced cultures compared to control cultures grown in the absence of γbb (Fig. 2*B* and Dataset S1). As expected from our previous cell-based activity experiments, expression of these genes was also up-regulated in cells grown with dl-carnitine, but not with GABA (*SI Appendix*, Fig. S2 and Dataset S1). The most differentially up-regulated genes are colocalized in the *E. timonensis* SN18 genome within a region we designate the γbb utilization (*bbu*) gene cluster (Fig. 2*C*). This six-gene cluster encodes a predicted flavin-dependent acyl-CoA dehydrogenase (*bbuA*), two class III CoA transferases (*bbuB* and *bbuC*), a di-[4Fe-4S]-cluster ferredoxin (*bbuD*), a betaine/carnitine/choline transporter (*bbuE*), and an acyl-CoA thioesterase (*bbuF*). The second set of genes up-regulated in γbb-induced cells encodes for predicted riboflavin biosynthesis enzymes (Dataset S1), which likely support cofactor biosynthesis for the enzyme encoded by *bbuA*. The highly specific transcriptional response to γbb suggested that the *bbu* gene cluster was involved in γbb metabolism. In addition, the presence of a gene encoding a transporter for trimethylammonium-containing compounds (*bbuE*) was consistent with this hypothesis.

### The CoA Transferases BbuB/C Initiate γbb Metabolism via Formation of a γbb-CoA Thioester.

**Fig. 3.**
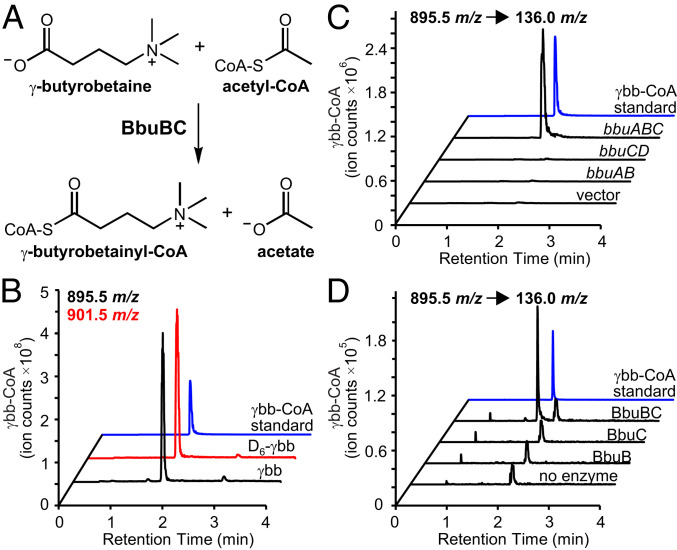
γbb-CoA is the first intermediate in γbb metabolism. (*A*) Chemical reaction catalyzed by the BbuB and BbuC proteins. (*B*) LC–MS extracted ion chromatograms of γbb-CoA ([M+H]^+^ = 895.5 *m/z*) and D_6_-γbb-CoA ([M+H]^+^ = 901.5 *m/z*) from extracts of *E. timonensis* cell suspensions incubated for 40 min with γbb (black) or D_6_-γbb (red), respectively, compared to a γbb-CoA standard (blue). (*C*) LC–MS/MS selected ion chromatograms (SIC) of the 136.0 *m/z* fragment ion of γbb-CoA produced from a 1-h incubation of γbb and acetyl-CoA with crude lysate of *E. coli* constitutively expressing *bbu* genes or empty vector. (*D*) LC–MS/MS SIC of the 136.0 *m/z* fragment ion of γbb-CoA produced from a 1-h incubation of γbb and acetyl-CoA with purified recombinant proteins. *SI Appendix*, Fig. S3 shows relative amounts of γbb-CoA from three biological replicates of experiments described for *C* and *D*.

Based on the predicted annotations of the *bbu* genes, we hypothesized that γbb would be initially activated for subsequent TMA elimination through formation of a CoA thioester. The conversion of a carboxylic acid to a thioester is a common first step in many metabolic pathways as it lowers the p*K*_a_ of the C_α_-protons, facilitating further reactions (26, 27). Indeed, in resting cell suspensions incubated with γbb, a metabolite was detected by liquid chromatography–mass spectrometry (LC–MS) with a peak retention time and mass-to-charge ratio (895.5 *m/z*, [M+H]^+^) matching those of a γbb-CoA standard (Fig. 3*A* and *SI Appendix*, Fig. S3). The origin of this metabolite was confirmed by incubating cell suspensions with deuterium-labeled 2,2,3,3,4,4-D_6_-γbb (D_6_-γbb), which led to an increase of 6 Da (901.5 *m/z*, [M+H]^+^) for the assigned γbb-CoA peak (Fig. 3*A* and *SI Appendix*, Fig. S3). These results support the hypothesis that γbb metabolism proceeds via a CoA thioester intermediate.

We next investigated whether two CoA transferases encoded by the *bbu* gene cluster, BbuB and BbuC, are directly involved in γbb-CoA production. These proteins both belong to the class III CoA transferase family but only share 30% amino acid sequence identity with one another. Indeed, γbb-CoA was detected from incubations of γbb with crude lysate of *E. coli* cells constitutively expressing the first three genes of the cluster (*bbuABC*) (Fig. 3*B*). Interestingly, γbb-CoA was not detected when either of the two CoA transferase genes was expressed alone (Fig. 3*B*). Production of γbb-CoA was also observed when recombinant, purified BbuB and BbuC were added together with γbb to lysate of *E. coli* transformed with empty vector (*SI Appendix*, Fig. S3) but, again, only when both proteins were present. The requirement for both CoA transferase proteins was also confirmed in vitro. In these experiments, the BbuB and BbuC proteins together generated γbb-CoA from γbb and an appropriate acyl-CoA donor substrate (Fig. 3*C*, *SI Appendix*, Fig. S3).

Based on these biochemical activity results, we proposed that the BbuB and BbuC form a functional hetero-oligomeric complex. To test our hypothesis, we conducted a pull-down affinity chromatography experiment using C-terminal streptavidin-tagged BbuB (BbuB-Strep) and N-terminal six histidine-tagged BbuC (BbuC-His). Lysates from separate heterologous expressions of each protein in *E. coli* were combined and loaded onto Ni^2+^-NTA resin to bind the BbuC-His protein, after which the resin was washed with buffers containing increasing concentrations of imidazole. We predicted that if the BbuB and BbuC proteins form a stable complex, high concentrations of imidazole would be required to displace the BbuB-Strep protein along with the BbuC-His protein. Western blot analysis of elution fractions using a streptavidin-specific antibody demonstrated that the BbuB-Strep protein was present in fractions eluted with high concentrations (250 mM) of imidazole (*SI Appendix*, Fig. S4). In contrast, control experiments without the BbuC-His protein showed BbuB-Strep protein only in the initial wash fractions with low concentrations (25 mM) of imidazole (*SI Appendix*, Fig. S4). These results provide initial evidence that BbuB and BbuC form a stable hetero-oligomeric complex, although the nature of the complex (e.g., stoichiometry, catalytic roles of each monomer) remains to be determined.

We next sought to determine the substrate specificity of the BbuB/C enzyme reaction. Various short-chain fatty acyl-CoAs (acetyl-, butyryl-, propionyl-, and crotonyl-CoA) were tested as CoA-donating substrates. While all compounds resulted in production of γbb-CoA, acetyl-CoA was the preferred cosubstrate (*SI Appendix*, Fig. S3). This preference for acetyl-CoA in vitro, however, does not preclude the use of an alternative donor in vivo depending on availability or requirements for energy conservation (e.g., CoA recycling via butyryl-CoA). We also tested dl-carnitine and GABA as alternative CoA acceptors in vitro with BbuB/C and acetyl-CoA as the donor. However, neither of the presumed products, carnitinyl-CoA or γ-aminobutyryl-CoA, was detected by LC–MS/MS under the reaction conditions tested (*SI Appendix*, Fig. S3). The high selectivity of the CoA transferases BbuB and BbuC for γbb directly connects the *bbu* gene cluster to γbb metabolism.

### The Flavoenzyme BbuA Catalyzes Elimination of TMA from γbb-CoA.

The key step in TMA production from γbb is breaking an unactivated C–N bond. None of the *bbu* genes is predicted to encode homologs of known enzymes that catalyze C–N bond cleavage and release TMA [e.g., choline TMA-lyase (28), glycine betaine reductase (29), ergothionase (30)], indicating that this pathway uses a distinct enzyme for this critical step. We identified the predicted acyl-CoA dehydrogenase-like enzyme BbuA as the most likely candidate for this reaction. Enzymes belonging to the acyl-CoA dehydrogenase family are flavin-dependent oxidoreductases that act on acyl-CoA thioester substrates, typically installing or removing an α,β-unsaturation through two-electron oxidation or reduction reactions, respectively (31). We envisioned that BbuA might instead use a flavin cofactor in a cryptic radical mechanism to activate γbb-CoA for TMA elimination from the C4 position. This proposed reaction resembles the C4 elimination of water from 4-hydroxybutyryl-CoA catalyzed by the flavin adenine dinucleotide (FAD)-dependent enzyme 4-hydroxybutyryl-CoA dehydratase (4HBD) involved in bacterial GABA and succinate metabolism (32). While BbuA and 4HBD are predicted to have a similar structure and harbor a flavin cofactor, they share very minimal sequence identity (∼10%).

**Fig. 4.**
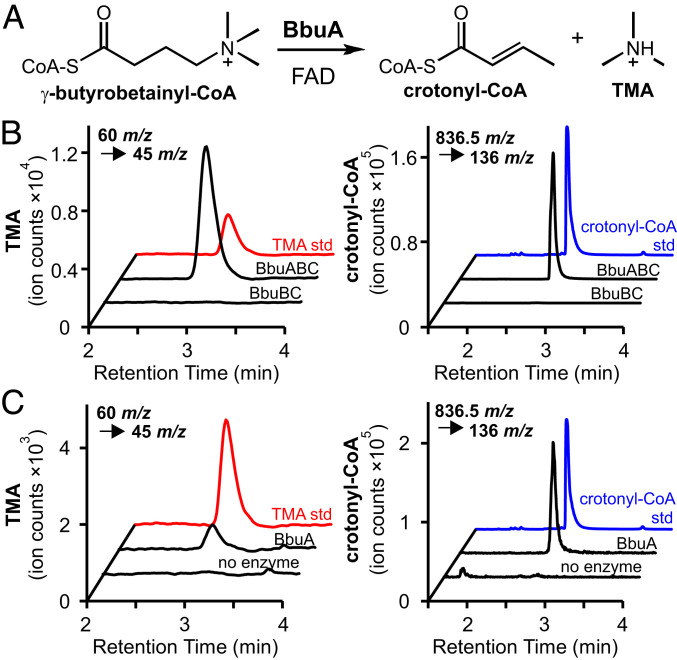
The FAD-dependent enzyme BbuA generates TMA. (*A*) Chemical reaction catalyzed by BbuA. (*B*) LC–MS/MS SIC of the precursor-fragment ion pairs of TMA (*Left*) and crotonyl-CoA (*Right*) produced from 1-h reactions containing γbb, acetyl-CoA, BbuB, BbuC, and BbuA or FAD (no enzyme). (*C*) LC–MS/MS SIC of the precursor-fragment ion pairs of TMA (*Left*) and crotonyl-CoA (*Right*) produced from 1-h reactions containing γbb-CoA with BbuA or FAD (no enzyme). *SI Appendix*, Fig. S5 shows quantification of TMA concentration and relative amounts of crotonyl-CoA from three biological replicates of experiments described for *B* and *C*.

Initial attempts to access soluble BbuA protein via heterologous expression in *E. coli* were unsuccessful. Soluble BbuA protein was only obtained under the following conditions: 1) coexpression with the native GroEL-ES proteins from *E. timonensis* SN18, 2) addition of exogenous riboflavin to the growth medium, and 3) addition of excess FAD during cell lysis. Crude lysates of *E. coli* cells overexpressing the *bbuABC* genes under these conditions demonstrated TMA production when incubated anaerobically with γbb and acetyl-CoA (*SI Appendix*, Fig. S5). Purified BbuA containing oxidized FAD was then tested for activity in vitro when incubated with γbb, acetyl-CoA, and the CoA transferases BbuBC. Robust TMA production was observed after a 1-h incubation without addition of redox mediators (Fig. 4*B*), demonstrating that these reaction components are sufficient to catalyze multiple turnovers of γbb. To probe whether the FAD cofactor is essential for BbuA activity, chemically reduced protein was tested for TMA production, as complete exclusion of FAD from the protein preparation led to protein insolubility. When BbuA was reduced with excess sodium dithionite, TMA production was severely diminished (*SI Appendix*, Fig. S5), suggesting that the FAD cofactor is required for activity and serves a redox role in the reaction. Finally, γbb-CoA was tested directly as the substrate for BbuA in vitro. This reaction also resulted in TMA production (Fig. 4*C*), demonstrating that BbuA alone is sufficient to catalyze TMA elimination in vitro. These results demonstrate that BbuA is the critical TMA-generating enzyme in O_2_-independent γbb metabolism by *E. timonensis*.

Next, we sought to determine the coproduct of the BbuA-catalyzed reaction. We predicted that crotonyl-CoA would be generated upon elimination of TMA from γbb-CoA by analogy to 4-hydroxybutyryl-CoA dehydration in GABA/succinate metabolism (32). Indeed, crotonyl-CoA was detected by LC–MS/MS in both the *E. coli* crude lysate incubations (*SI Appendix*, Fig. S5) and the in vitro assays with purified BbuA (Fig. 4). Both the retention time and *m/z* of the detected metabolite were consistent with a crotonyl-CoA standard (*SI Appendix*, Fig. S5). The production of crotonyl-CoA supports the proposed redox-neutral TMA elimination from γbb-CoA (Fig. 4). Together, the gain-of-function experiments in *E. coli* and in vitro activity assays demonstrate that the *bbu* gene cluster is responsible for TMA production from γbb and that the three genes *bbuABC* are necessary and sufficient to confer this function in *E. coli* and in vitro.

### *E. timonensis* Metabolizes γbb for Anaerobic Respiration and Carbon Acquisition.

**Fig. 5.**
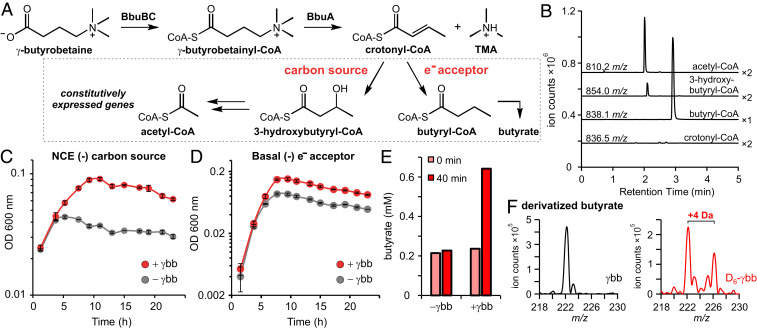
γbb-derived crotonyl-CoA is further metabolized by *E. timonensis* for carbon and respiration. (*A*) Complete metabolic pathway using γbb as a carbon source and electron acceptor. (*B*) LC–MS/MS SICs of parent ions with 136.0 *m/z* fragments that correspond to expected products of crotonyl-CoA metabolism from extracts of *E. timonensis* SN18 cell suspensions incubated with γbb for 40 min. Chromatogram magnification is indicated next to each trace. (*C* and *D*) Anaerobic growth curves of *E. timonensis* SN18 in minimal media lacking (*C*) carbon sources or (*D*) electron acceptors with (red) or without (black) γbb. Error bars represent SD from the mean of three biological replicates. (*E*) Concentrations of derivatized butyrate detected from extracts of *E. timonensis* cell suspensions incubated for 0 (pink) or 40 (red) min in the presence or absence of 1 mM γbb. Bars show the average of two biological replicates. (*F*) Mass spectra of derivatized extracts (unlabeled butyrate [M+H]^+^ = 222 *m/z*) of *E. timonensis* cell suspensions incubated for 40 min with γbb (black) or D_6_-γbb (red).

Although crotonyl-CoA was produced in lysate and in vitro reactions containing BbuA, crotonyl-CoA did not accumulate in resting cell suspensions of *E. timonensis* SN18 incubated with γbb. Instead, we detected the known products of bacterial crotonyl-CoA metabolism: 3-hydroxybutyryl-CoA, acetyl-CoA, and butyryl-CoA (Fig. 5*B*). Inspection of their mass spectra showed deuterium incorporation when D_6_-γbb was used, confirming their origin from γbb (*SI Appendix*, Fig. S6). These results suggest that crotonyl-CoA generated from γbb is further metabolized by *E. timonensis*. Notably, the *bbu* gene cluster does not encode any enzymes known to be involved in crotonyl-CoA metabolism (33). However, protein homologs of enoyl-CoA hydratase, 3-hydroxybutyryl-CoA dehydrogenase, and butyryl-CoA dehydrogenase are encoded by constitutively expressed genes located elsewhere in the *E. timonensis* SN18 genome (Dataset S2). To test whether they could account for the observed crotonyl-CoA metabolism, crotonyl-CoA was incubated with cell lysates of *E. timonensis* SN18 grown in the presence or absence of γbb. In both cases, crotonyl-CoA was converted to 3-hydroxybutyryl-CoA during an hour incubation (*SI Appendix*, Fig. S7). Longer incubations led to accumulation of acetyl-CoA and low levels of butyryl-CoA (*SI Appendix*, Fig. S7). However, these downstream conversions in the assay were likely limited by the cofactor concentrations present in the lysate and thus do not represent the native partition of the end-products from crotonyl-CoA metabolism. These results demonstrate that the final steps of γbb metabolism in *E. timonensis* SN18 are known transformations of crotonyl-CoA, catalyzed by constitutively produced enzymes encoded outside of the *bbu* gene cluster (Fig. 5*A*).

Some species of Clostridia can use crotonyl-CoA metabolism for anaerobic respiration via reduction to butyryl-CoA and as a carbon and energy source through conversion to acetyl-CoA (Fig. 5*A*) (34, 35). We examined whether γbb metabolism via crotonyl-CoA would enhance the growth of *E. timonensis* SN18 in media conditions with limited electron acceptors or carbon sources. In both culture conditions, the growth yield was markedly enhanced with γbb supplementation (Fig. 5 *C* and *D*) and the growth rate was consistent with the rates of γbb depletion and TMA production (*SI Appendix*, Fig. S8). These results support a key role for crotonyl-CoA as an intermediate in γbb metabolism and indicate a physiological role for this activity in *E. timonensis*.

Due to the prominent role of butyrate in the human gut, we next investigated whether free butyrate was produced from *E. timonensis* γbb metabolism. Using a carboxylate-derivatization method and LC–MS, elevated levels of butyrate were detected in suspensions of γbb-induced cells incubated with γbb compared to suspensions of noninduced cells (Fig. 5*E* and *SI Appendix*, Fig. S9). In addition, deuterium incorporation into butyrate was observed when D_6_-γbb was used (Fig. 5*F*). Analysis of the mass spectrum revealed that the D_6_-isotopolog was not present; rather the +4 Da species was the primary isotopolog detected (Fig. 5*F* and *SI Appendix*, Fig. S9). The loss of two deuteria in the butyrate product is consistent with a crotonyl-CoA precursor. To rule out the possibility that the loss of label resulted from exchange of the acidic C_α_-deuterons with protons from solution, a control experiment was performed incubating perdeuterated D_7_-butyrate with cell suspensions. The mass spectrum of butyrate from this experiment showed minimal deuterium exchange with solvent protons during the same incubation period (*SI Appendix*, Fig. S9). In addition, γbb-CoA detected from incubations with D_6_-γbb showed minimal deuterium loss (*SI Appendix*, Fig. S9). These control reactions support the proposal that γbb metabolism in *E. timonensis* proceeds through the α,β-unsaturated crotonyl-CoA intermediate and generates free butyrate in resting cell suspensions. To further investigate whether butyrate was the major end-product of γbb metabolism in actively growing *E. timonensis*, we quantified derivatized butyrate by LC–MS from cultures grown with D_6_-γbb in rich medium and the two types of minimal media described above. Deuterium-labeled butyrate accounted for only 8.5% of the total butyrate produced in rich media but up to 34% and 40% of the total butyrate produced in minimal media with limited electron acceptors and carbon sources, respectively. However, in each medium, the deuterium-labeled butyrate only amounted to ∼5% of the original γbb, indicating that butyrate is not the major final product of this metabolism and thus is not likely to contribute substantially to overall levels of butyrate produced in the gut.

### The *bbu* Gene Cluster Predicts γbb Metabolism in Bacterial Isolates.

**Fig. 6.**
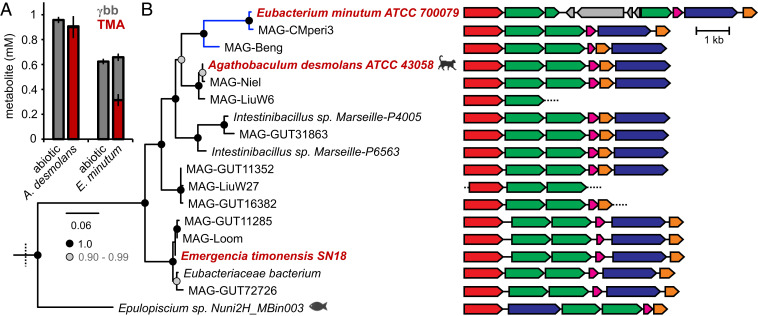
Cultured and uncultured host-associated Clostridiales bacteria possess *bbu*-like gene clusters indicative of γbb metabolism. (*A*) TMA and γbb detected by LC–MS/MS from pure cultures of *A. desmolans* and *E. minutum* grown in rich media supplemented with γbb. Error bars represent the SD from the mean of three biological replicates. (*B*) BbuA clade of a maximum-likelihood phylogenetic tree and genomic context of BbuA homologs. aBayes branch supports are shaded based on the legend. Red labels indicate experimentally validated γbb-metabolizing bacteria. Blue branches indicate an isolate or MAG originating from an oral sample. Dashed lines indicate the end of a contig. MAG identifiers are listed in Dataset S3.

As noted previously, *E. timonesis* is the only bacterial species known to metabolize γbb to TMA under anaerobic conditions. Having discovered the gene cluster responsible for this activity, we searched for homologous gene clusters in other sequenced bacterial genomes. We identified *bbu*-like gene clusters in four host-associated, cultured bacteria belonging to the order Clostridiales. To examine whether the *bbu* genes are diagnostic of γbb metabolism, two commercially available strains, the human oral isolate *Eubacterium minutum* American Type Culture Collection (ATCC) 700079 and the feline gut isolate *Agathobaculum desmolans* ATCC 43058 (previously *Eubacterium desmolans* or *Butyricoccus desmolans*), were tested for this activity. Both strains converted γbb to TMA in anaerobic cultures (Fig. 6*A*), demonstrating that the presence of the *bbu* gene cluster predicts the capacity for anaerobic γbb metabolism. The rarity of this gene cluster in sequenced genomes of cultured bacteria motivated us to search uncultured bacterial genomes that had been assembled from human metagenomes (i.e., metagenome-assembled genomes [MAGs]) (36, 37). We identified homologous *bbu* gene clusters in 11 uncultured, human-associated MAGs, all of which belong to the order Clostridiales (Fig. 6*B* and Dataset S3). Two of the uncultured bacterial genomes were assembled from human oral samples, while the remaining genomes harboring the *bbu* gene cluster were assembled from human stool samples. The presence of the *bbu* gene cluster in multiple uncultured gut bacteria suggests that γbb metabolism is underrepresented among cultured isolates and that *E. timonensis* may not be solely responsible for this metabolism in the human gut. Finally, we searched for the presence of the *cai* genes responsible for metabolizing l-carnitine to γbb in these *bbu*-containing bacterial genomes. However, none of these genomes possess homologs of known *cai* genes, indicating that these γbb-metabolizing bacteria are incapable of the full conversion of l-carnitine to TMA.

### Presence of *bbu* Genes in Human Gut Metagenomes Is Correlated with Lower γbb Levels.

**Fig. 7.**
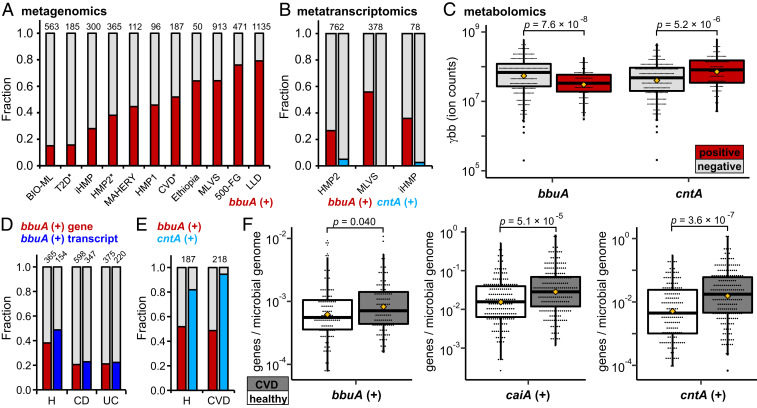
Anaerobic γbb metabolism genes are widely distributed and expressed in the human gut microbiota. (*A*) Fraction of stool metagenomes that are positive (red) and negative (gray) for the *bbuA* gene from healthy donors in human studies (Dataset S4). Study sample size (*N*) is indicated above each bar. Asterisks indicate that only healthy donors from those projects were included in this analysis. (*B*) Fraction of stool metatranscriptomes that are positive and negative for *bbuA* or *cntA*. Study sample size (*N*) is indicated above each bar. (*C*) Correlation between the presence of *bbuA* or *cntA* genes in metagenomes and γbb metabolite levels in HMP2 stool samples. Mean values are represented by yellow diamonds, and *P* values were determined using the Mann–Whitney *U* test. (*D*) Fraction of samples positive for the *bbuA* gene or transcripts in HMP2 IBD cohorts compared to healthy controls. Study sample size (*N*) is indicated above each bar. (*E*) Fraction of samples positive for the *bbuA* or *cntA* gene in a CVD cohort compared to healthy controls. Study sample size (*N*) is indicated above each bar. (*F*) Correlations between the presence of the *bbuA*, *caiA*, or *cntA* genes in CVD subjects (grey) compared to healthy controls (white). Mean values are represented by yellow diamonds, and *P* values were determined using the Mann–Whitney *U* test.

Motivated by the presence of the *bbu* gene cluster in uncultured gut bacteria and the implications for γbb metabolism in human health, we assessed the prevalence of the *bbu* gene cluster in publicly available human metagenomic datasets. Our analysis used the *bbuA* gene as a representative of the full cluster in stool metagenomes from healthy adult populations. We detected hits for the *bbuA* gene in samples from every cohort, but the proportion of *bbuA*-positive samples varied substantially between cohorts, ranging from 15% to 85% positive (Fig. 7*A*). We also quantified the abundance of the *bbuA* gene in *bbuA*-positive samples and found that it was consistent across cohorts at 1:1,000 genes per microbial genome, with a range of 1:10^2^ to 1:10^4^ genes per microbial genome (*SI Appendix*, Fig. S10). Since diet has been reported as an important factor in TMA production capacity of the microbiota, we examined whether *bbuA* gene presence or abundance was correlated with diet. However, neither *bbuA* gene presence nor abundance was correlated with self-reported omnivorous or vegetarian donors from the BIO-ML cohort (*SI Appendix*, Fig. S11). Overall, the distribution profile of *bbu* genes suggests that γbb-metabolizing bacteria are frequently present in the human gut microbiota but are low-abundance members of the community.

To provide further support for the relevance of the *bbu* pathway in the human gut, we evaluated gene expression using metatranscriptomic data from three cohorts: HMP2 (38), iHMP (39), and MLVS (40). We detected *bbuA* transcripts in 27% to 56% of samples in these cohorts (Fig. 7*B*), suggesting that the *bbu* pathway is expressed in the human gut. Transcripts of the *bbuA* gene were detected in the same samples that were positive for the *bbuA* gene but also in a subset (16% to 26%) of *bbuA*-negative samples (*SI Appendix*, Fig. S12). This observation suggests that the *bbuA* gene abundance was too low to be detected by the metagenomic sequencing but that the transcript levels were high enough to be captured by the read depth of the metatranscriptomic sequencing. Notably, very few samples in these cohorts (0% to 4%) were positive for *cntA* transcripts (Fig. 7*B* and *SI Appendix*, Fig. S12), consistent with previously reported analyses (41). Thus, the metatranscriptomic data suggest that the *bbu* pathway is more likely to be functionally operant in the human gut than the O_2_-dependent pathway mediated by *cntA*.

Next, we examined three cohorts with corresponding stool metabolomics data [HMP2 (38), BIO-ML (42), and PRISM (43)] to evaluate whether the presence of *bbuA* gene or transcripts is associated with changes in metabolite levels. In each of the three independent cohorts, the levels of γbb were lower in samples that were positive for the *bbuA* gene compared to those that were negative (Fig. 7*C* and *SI Appendix*, Fig. S13). In the HMP2 cohort, the samples positive for *bbuA* transcripts were also correlated with lower γbb levels (*SI Appendix*, Fig. S14). The *bbuA* gene, however, was not associated with stool carnitine or butyrate levels (*SI Appendix*, Fig. S15). In contrast, the presence of the *cntA* gene was correlated with higher or equivalent γbb levels, rather than lower, when compared to *cntA*-negative samples (Fig. 6*C* and *SI Appendix*, Fig. S13). These results suggest that the *bbu* gene cluster may be primarily responsible for γbb consumption in the human gut.

Finally, we evaluated whether differences in the presence or abundance of the *bbuA* gene are correlated with human disease. Although TMA production is not known to be a factor in inflammatory bowel disease (IBD), we noticed that the *bbuA* gene was detected in fewer samples from donors with Crohn’s disease and ulcerative colitis compared to healthy controls in both HMP2 (38) and PRISM (43) cohorts (Fig. 7*D* and *SI Appendix*, Fig. S16). The same trend was observed when analyzing *bbuA* transcripts in the HMP2 study (Fig. 7*D*). However, there was no difference in gene abundance between the IBD and healthy cohorts (*SI Appendix*, Fig. S16). Next, we analyzed metagenomes of a CVD cohort from China (44). The proportion of *bbuA*-positive samples was not different between healthy and CVD groups (Fig. 7*E*), but the gene abundance in *bbuA*-positive samples was modestly higher in CVD donors compared to healthy controls (Fig. 7*F*). A more significant elevation was found in the *caiA* gene abundance in the CVD cohort compared to the healthy group (Fig. 7*F*), suggesting that the conversion of l-carnitine to γbb may be a more relevant biomarker for CVD than the *bbu* gene cluster. Consistent with previous reports (41), we also detected a higher prevalence and abundance of the *cntA* gene in CVD samples compared with healthy controls from this study (Fig. 7 *E* and *F*). However, the lack of correlation between *cntA* gene presence and expression observed in other cohorts raises questions about the functional relevance of this finding.

## Discussion

Gut microbial production of TMA from dietary l-carnitine has been recognized for decades, but our knowledge of the reactions, enzymes, and organisms involved in the O_2_-independent pathway has remained incomplete. We used RNA-seq to identify a substrate-inducible *bbu* gene cluster that is responsible for anaerobic γbb metabolism in the gut microbe *E. timonensis*. We demonstrated that the enzymes encoded within the *bbu* gene cluster are necessary and sufficient to convert γbb to TMA in vitro and for gain-of-function in *E. coli*. With the discovery of the genetic basis for anaerobic γbb metabolism, we then determined the chemical steps involved, identified other gut microbes with this function, and evaluated the relevance of this microbial activity in human populations.

The pathway for anaerobic metabolism of γbb provides an interesting parallel to GABA metabolism by gut microbes (45). Given the structural similarity of the two molecules, which differ by only the *N*-methylation of γbb, the use of comparable pathways for their catabolism is perhaps not surprising. Analogous chemical steps, including substrate activation via CoA formation and C4 elimination, occur in both pathways to yield the common product crotonyl-CoA. This intermediate is then processed by the bacteria as a carbon and energy source or as an electron acceptor. Yet there are notable distinctions in the substrate activation steps and key lyase enzymes of each pathway. Conversion of the carboxylate to the thioester lowers the p*K*_a_ of the C_α_-protons (26, 27), facilitating elimination of the C4 hydroxyl or trimethylammonium group in the 4HBD- or BbuA-catalyzed reaction, respectively. Due to its positive charge, the trimethylammonium moiety of γbb-CoA is a good leaving group for C4 elimination. At physiological pH, the primary amine of GABA would also be protonated and positively charged (46), which could enable an analogous direct elimination from a hypothetical γ-aminobutyryl-CoA intermediate. Interestingly, GABA-metabolizing bacteria instead convert the amine into a hydroxyl group, which is a worse leaving group, prior to formation of the CoA thioester and C4 elimination. This biochemical pathway may be the result of repurposing the 4HBD enzyme from succinate metabolism in bacteria and archaea (47–49), which proceeds via a 4-hydroxybutyryl-CoA intermediate. The flavin-dependent lyase enzymes, 4HBD and BbuA, that catalyze the key C4 elimination step in these pathways both belong to the broad structural superfamily of acyl-CoA dehydrogenases (31). This generic functional annotation implies two-electron redox chemistry using a flavin cofactor. However, 4HBD diverges from this canonical function and instead is proposed to use a radical-based mechanism (50) wherein the flavin cofactor performs single-electron chemistry to mediate water elimination. BbuA could use a similar mechanistic strategy for TMA elimination; however, these two proteins have negligible (<10%) sequence identity to one another. Thus, it remains an open question as to whether they use distinct mechanisms to catalyze seemingly analogous, chemically challenging C4-elimination reactions. The mechanism of the TMA-lyase reaction catalyzed by BbuA will be the subject of future studies.

Our current understanding of BbuA reveals that it is unique compared to the other known TMA-producing enzymes. The l-carnitine monooxygenase CntA uses a metallocofactor to activate dioxygen for substrate hydroxylation, generating a chemically unstable product that breaks down to eliminate TMA. Conversely, BbuA directly catalyzes the C–N bond cleavage step. The enzyme glycine betaine reductase catalyzes an overall two-electron reduction of glycine betaine to produce TMA, employing protein-substrate covalent adducts via (seleno)cysteine residues (29). In contrast, BbuA catalyzes a redox-neutral transformation and requires an oxidized flavin cofactor. The choline TMA-lyase CutC is a glycyl radical enzyme which uses protein-based radicals to initiate catalysis (28). CutC is proposed to generate a carbon-centered substrate radical intermediate that undergoes a spin center shift and promotes direct 1,2-elimination of TMA (51). The BbuA enzyme could share a similar radical-based mechanistic logic with CutC but, instead of protein-based radicals, may use a flavin cofactor for radical generation. However, the chemical differences between the γbb and choline substrates—the aliphatic carbon chain attached to the trimethylammonium in γbb compared to the vicinal hydroxyl group in choline that actively participates in TMA elimination—will necessitate mechanistic divergence. Intriguingly, the mechanism employed by CutC could theoretically operate using l-carnitine as the substrate, as it also has a vicinal alcohol, yet such a hypothetical reaction is not known to exist.

Anaerobic γbb metabolism is a microbial function found in only a select group of human gut bacteria. Prior to this work, *E. timonensis* was the only organism known to perform this transformation and, correspondingly, was found in high abundance in high-TMAO producers from a human carnitine-challenge study (52). However, *E. timonensis* was only detected in 25% of individuals from that group (52), suggesting that additional gut bacteria possess this metabolic activity. Indeed, we have discovered that the *bbu* gene cluster is present in other related gut bacteria. The *bbu* gene cluster was found exclusively in obligate anaerobic bacteria of the Clostridiales order, but it is not ubiquitous within this taxonomic group. This observation contrasts with choline metabolism, which is widely distributed across different gut microbial phyla and classes (53). In addition, the *bbu* gene cluster is only found in host-associated bacteria. While most *bbu*-encoding bacteria were isolated from human stool, a few bacteria originated from the human oral cavity. Bacteria that inhabit the gastrointestinal tract of other animals (i.e., cat and fish) were also found to possess *bbu* gene clusters, suggesting a broader range of hosts. Anaerobic TMA production from γbb has not been reported outside of host-associated contexts, consistent with our finding that the *bbu* gene cluster is not found in environmental bacteria. Thus, a select group of anaerobic gut bacteria appear to possess a specialized metabolism that supports their growth in this niche.

Our discovery of the genetic basis for γbb metabolism confirms the proposal that the anaerobic conversion of l-carnitine to TMA is an interspecies metabolic pathway. Our analyses show that bacteria possessing the *bbu* pathway to produce TMA from γbb do not have the *cai* operon required to convert l-carnitine to γbb and vice versa. This observation could explain why γbb-metabolizers inhabit host environments where l-carnitine is abundant due to dietary consumption and where other bacteria can convert it to γbb. The inability of γbb-metabolizing bacteria to generate γbb from l-carnitine highlights the importance of cross-feeding in the gut microbiota. There are now many examples of microbial cometabolism in the gut (54), including polysaccharide utilization (55), lignin degradation (56), and even drug metabolism (57). Since TMA can only be produced anaerobically when both l-carnitine and γbb utilizing organisms are present, an investigation into their co-occurrence will be important to deconvolute the contribution of anaerobic l-carnitine metabolism to TMA generation in the human gut.

Our initial bioinformatic analyses indicate that the *bbu* pathway producing TMA is likely functionally important in the human gut. Using the *bbuA* gene as a marker for anaerobic γbb metabolism, we find that this activity is widely distributed in human stool metagenomes. We also detect *bbuA* transcripts in human stool sequencing data, demonstrating that these genes are expressed in the gut environment. Furthermore, the presence of the *bbuA* gene and transcripts is correlated with lower levels of γbb in stool, suggestive of active γbb consumption in subjects possessing this gut bacterial pathway. Conversely, the *cntA* gene is not correlated with changes in stool metabolite levels, nor is it highly expressed in the human gut. This lack of expression could perhaps indicate dioxygen-dependent regulation of *cntA*. Intriguingly, 79% of the HMP2 samples (30/38) that were positive for *cntA* transcripts were from donors with IBD, suggesting that factors associated with inflammation, such as increased O_2_ levels, may induce expression of *cntA*. An independent study also concluded that the *cntA* gene is not expressed in healthy human gut microbiomes (41). Our analysis replicates their reported results using two of the same cohorts (MLVS and iHMP) and adds the larger HMP2 cohort that was since published. Together, our bioinformatic analyses using the *bbuA* gene as a marker for the later steps in anaerobic l-carnitine metabolism suggest that the O_2_-independent pathway, rather than the O_2_-dependent pathway, is more relevant for TMA production from l-carnitine in the anoxic gut environment. This conclusion advises caution when inferring functional relevance from gene presence alone and highlights the importance of considering how environmental factors can influence microbial metabolic functions.

Our evaluation of anaerobic γbb metabolism in the human gut microbiota suggests that this pathway may be associated with disease states. In IBD cohorts, the lower prevalence of the *bbuA* gene and transcripts could reflect a decrease in O_2_-intolerant bacteria, such as *bbu*-encoding Clostridiales, due to elevated O_2_ levels caused by inflammation. This trend has been noted for other microbial functions that are restricted to obligate anaerobes, for example, metabolism of cholesterol to coprostanol by uncultured *Clostridia* (58). Although TMA production has not been linked to IBD pathology, this observation demonstrates how environmental conditions can influence metabolic functions. On the other hand, the strong connection between TMA production from l-carnitine and CVD motivated our targeted analysis of γbb metabolism in this disease. We did not find a significant difference in the prevalence of *bbu* genes in CVD donors compared to healthy controls, but we did observe that the abundance of *bbu* and *cai* genes was elevated in CVD donors. However, our analysis of paired metagenomic and metatranscriptomic datasets (not associated with CVD) highlights challenges in drawing conclusions from metagenomic data alone. We found that the presence or absence of the *bbu* genes in metagenomic data does not always reflect the presence or absence of *bbu*-encoding organisms. For example, samples that are negative for *bbu* genes can be positive for *bbu* transcripts, suggesting that the *bbu*-expressing organisms are present but perhaps in too low of an abundance to be detected by the metagenomic sequencing read depth. Furthermore, we experimentally demonstrate that expression of the *bbu* gene cluster is regulated by γbb and l-carnitine in *E. timonensis*. These results together indicate that transcriptomics is a better reporter of metabolic function, particularly for low-abundance members of the microbiota like γbb-metabolizing bacteria.

A more comprehensive evaluation of the contribution of anaerobic γbb metabolism to overall TMA levels in human populations should integrate dietary information and host serum metabolites with multi-omic analyses. Dietary studies have demonstrated a greater capacity for the gut microbiota from omnivorous donors to produce TMA from l-carnitine compared to vegans or vegetarians (7, 13, 25, 52). Our analysis of the *bbuA* gene in self-reported dietary groups did not reveal differences in prevalence or abundance. However, a more thorough analysis of co-occurrence of l-carnitine and γbb metabolism in controlled dietary cohorts may reveal differences. Diet may also be a complicating factor to consider in meta-analyses of disease cohorts. Finally, although our results show that the *bbuA* gene and transcripts are correlated with lower γbb levels, implying increased production of TMA from this substrate, we were unable to evaluate TMA and TMAO levels from stool metabolomics in these human populations because they are most reliably detected in serum, plasma, or urine. Demonstrating this correlation will be important to conclusively link anaerobic metabolism of l-carnitine–derived γbb to elevated systemic levels of TMA(O) in healthy and disease populations. Our discoveries set the groundwork for subsequent analyses to address this connection.

This work highlights the importance of enzyme discovery for understanding the chemical basis of gut microbial metabolite production, connecting metabolic activities with specific microbes, and analyzing gut microbial functions in human populations. We have uncovered a distinct enzymatic strategy to generate TMA from γbb in an O_2_-independent manner. The BbuA enzyme acts in a pathway that leads to the production of metabolites that are independently associated with human disease and health. We have discovered an additional important group of TMA-producing bacteria in the human gut microbiota. The identification of the *bbu* gene cluster in many uncultured bacteria highlights this portion of the microbiota as a rich source of underappreciated chemistry that can have important roles in human health. Finally, the identification and characterization of the *bbu* pathway fills an important gap in our knowledge of TMA production from l-carnitine in the anoxic human gut. With a more complete understanding of the metabolic network involved in TMA production, we can begin to dissect the dietary, microbial, and metabolic factors that lead to its generation and connection to human disease.

## Materials and Methods

### Bacterial Strains and Culture Conditions.

*E. timonensis* SN18 was purchased from Leibniz Institute DSMZ. *E. timonensis* SN18 culturing was performed in Hungate or Balch tubes (Chemglass Life Sciences) at 37 °C and set up in an anaerobic chamber (Coy Laboratory Products) under an atmosphere of 2% to 4% H_2_, 2% to 4% CO_2_, and N_2_ as the balance. Standard cultures were grown in peptone-yeast-glucose (PYG) medium, modified (medium recipe DSM 104, DSMZ Germany) that was sparged with N_2_ after autoclaving. Basal medium lacking electron acceptors was prepared as described previously (57), containing 1 g/L tryptone (trypticase peptone; BD Biosciences), 1 g/L yeast extract (BD Biosciences), 0.4 mM l-cysteine, 2.5 g/L NaHCO_3_, 1 g/L NaCl, 0.5 g/L MgCl_2_•6H_2_O, 0.2 g/L KH_2_PO_4_, 0.3 g/L NH_4_Cl, 0.3 g/L KCl, 0.015 g/L CaCl_2_•2H_2_O, 0.25 mL/L of 0.1% resazurin, and 1% ATCC vitamins and trace mineral solutions (ATCC). NCE medium lacking carbon sources was prepared as described previously (59), containing 4 g/L KH_2_PO_4_, 5 g/L K_2_HPO_4_, 3.5 g/L NaNH_4_PO_4_ 40 mM sodium fumarate dibasic, 1 mM MgSO_4_•7H_2_O, 0.1% casamino acids (VWR Life Science), and 1% ATCC vitamin and trace mineral solutions (ATCC). All chemicals were purchased from Sigma-Aldrich unless otherwise indicated.

### Chemical Synthesis of [*N*-(CD_3_)_3_]-γbb and D_6_-γbb.

The following general procedure was used for synthesis of both [*N*-(CD_3_)_3_]-γbb and D_6_-γbb, based on a published protocol (60). In an oven-dried round bottom flask under nitrogen, 1 molar equivalent of GABA (1 g, 9.7 mmol) or D_6_-GABA (92 mg, 0.8 mmol) and 4.6 molar equivalents of K_2_CO_3_ were dissolved in anhydrous methanol (0.1 M) and stirred at room temperature for 15 min. Next, 5.1 molar equivalents of CD_3_I (3.1 mL, 49.4 mmol) or CH_3_I (0.42 mL, 4.3 mmol) was added to the solution, which was stirred at room temperature for 3 d. The reaction mixture was then concentrated under vacuum. The residue was resuspended in chloroform. The solid was filtered under vacuum and washed twice with chloroform. The solid was dissolved in 10% aqueous HCl (0.5 M). The resulting solution was concentrated under vacuum and further dried under vacuum. The residue was triturated three times with acetone. The combined organic layers were concentrated under vacuum. Anhydrous tetrahydrofuran (THF; 0.1 M) was added and the solution was cooled to 0 °C. The solid was filtered under vacuum and washed with fresh THF to afford the desired products.

*N*-(CD_3_)_3_]-γbb was obtained as a white solid (160 mg, 1 mmol, 10% yield) with the following NMR features: ^1^H NMR (400 MHz, D_2_O) δ ppm 2.07 to 2.16 (m, 2H), 2.53 (q, 2H, *J* = 7.3 Hz), 3.37 (td, 2H, *J* = 8.3, 3.9 Hz); ^13^C NMR (100 MHz, D_2_O) δ ppm 75.2, 87.3, 109.8, 122.4, 232.4. The NMR data were in accordance with literature values (60).

D_6_-γbb was obtained as a yellow solid (76 mg, 0.4 mmol, 48% yield) with the following NMR features: ^1^H NMR (400 MHz, D_2_O) δ ppm 3.07 (s, 9H).

### Assays in *E. timonensis* Whole-Cell Suspensions.

*E. timonensis* SN18 was cultured anaerobically at 37 °C in 10 mL PYG-modified medium supplemented with 1 mM γbb, dl-carnitine, or GABA or with an equivalent volume of 1× phosphate-buffered saline (PBS). When the cultures reached an OD_600_ = 0.5, they were centrifuged at 1,500 × *g* for 10 min at 4 °C. In an anerobic chamber (Coy Laboratory Products) under an atmosphere of 2% to 4% H_2_, 2% to 4% CO_2_, and N_2_ as the balance, the cell pellets were resuspended in 1 mL anoxic 1× PBS and centrifuged at 1,500 × *g* for 10 min at 4 °C. The cell pellets were resuspended in 0.5 mL anoxic 1× PBS and incubated at room temperature for 1 h with 1 mM γbb, [*N*-(CD_3_)_3_]-γbb, D_6_-γbb, dl-carnitine, or GABA or with an equivalent volume of 1× PBS. Reactions were analyzed by LC–MS as described below using three different methods for γbb/TMA, CoA, and fatty acid detection.

### RNA Sample Preparation, Sequencing, and Data Analysis.

*E. timonensis* SN18 cultures were prepared in an anerobic chamber (Coy Laboratory Products), under an atmosphere of 2% to 4% H_2_, 2% to 4% CO_2_, and N_2_ as the balance, in Balch tubes containing 15 mL PYG-modified medium. Cultures were grown anaerobically at 37 °C to an OD_600_ = 0.7, when 0.15 mL of a 1 M O_2_-free stock solution of γbb prepared in 1× PBS was added (1 mM final concentration) or an equivalent volume of O_2_-free 1× PBS was added to triplicate cultures for each condition. In a separate experiment (*SI Appendix*, Fig. S2), when cultures reached an OD_600_ = 0.5, 0.15 mL of 1 M O_2_-free stock solutions of γbb, dl-carnitine, or GABA prepared in 1× PBS were added (1 mM final concentration) or an equivalent volume of O_2_-free 1× PBS was added to triplicate cultures for each condition. Cultures were grown anaerobically at 37 °C for an additional 45 min and then centrifuged at 3,320 × *g* for 10 min at 4 °C. Keeping the samples cold on ice, the supernatant was decanted and then removed entirely using a micropipette. Cell pellets were immediately resuspended in 0.5 mL cold TRIzol reagent (Thermo Fisher) and flash frozen in liquid N_2_. Samples were stored at −80 °C until further processing. RNA isolation, library preparation, sequencing, and analysis are described in the *SI Appendix*.

### Cloning, Heterologous Expression, and Protein Purification.

Construction of plasmids containing the *bbu* genes used for gain-of-function experiments and heterologous expression are described in detail in the *SI Appendix* and Dataset S5. Preparation of *E. timonensis* BbuA, BbuB, and BbuC proteins as well as the γbb:CoA ligase *S. meliloti* BcoAB are described in detail in the *SI Appendix*.

### Affinity Pull-Down Chromatography of the BbuB-Strep Protein.

Construction of the plasmid for production of C-terminal streptavidin-tagged BbuB and the affinity pull-down chromatography experiment are described in detail in the *SI Appendix*.

### Chemical Synthesis of Crotonyl-CoA.

Crotonyl-CoA was synthesized from the acid anhydride according to a published protocol (61) with modifications described in the *SI Appendix*.

### Enzymatic Synthesis of γbb-CoA.

A 1 mL solution of 0.02 mM *S. meliloti* BcoAB, 0.2 M MgCl_2_, 0.2 M adenosine triphosphate (ATP), 0.02 M CoA (CoALA Biosciences, Austin, TX), and 0.05 M γbb in 0.5 M Tris⋅HCl (pH 8.0) was incubated at room temperature for 30 min. The reaction was quenched with 1% acetic acid (final concentration) and incubated at −20 °C for 30 min. The solution was centrifuged at 16,000 × *g* for 30 min. The γbb-CoA product was purified by preparative HPLC using the same method as described for crotonyl-CoA. Fractions with absorption at 260 nm were collected and lyophilized. The product identity was confirmed by mass spectrometry.

### CoA Transferase Assays in *E. coli* Lysates.

*E. coli* MG1655 chemically competent cells were transformed with pET-proD (vector construction described in *SI Appendix*) plasmids containing *E. timonensis bbu* genes (Dataset S5). Transformed cells with kanamycin resistance were grown in 50 mL Luria Broth (LB) medium with 50 mg/L kanamycin at 37 °C with shaking (180 rpm) for 20 h, reaching an OD_600_ of 1 to 1.4. Culture aliquots of 15 mL were harvested by centrifugation at 3,220 × *g* for 15 min at 4 °C and cell pellets were frozen at −80 °C until further use. Cell pellets were resuspended with a volume of 50 mM potassium phosphate (pH 7.5) buffer, 300 mM NaCl, and 10% glycerol to achieve a normalized OD_600_ of 20. The cells were lysed on ice by sonication for a total of 1 min, with cycles of 2.5 s on and 10 s off. Reactions (0.05 mL total) containing 0.01 mL crude lysate, 10 mM acetyl-CoA (CoALA Biosciences, Austin, TX), and 25 mM γbb in 100 mM Tris⋅HCl (pH 8.0) buffer were incubated at room temperature for 1 h and analyzed by LC–MS using the CoA detection method (*SI Appendix*).

### TMA-Lyase Assays in *E. coli* Lysates.

*E. coli* MG1655 (DE3) chemically competent cells were cotransformed with a pET28a plasmid containing *E. timonensis bbu* genes and the pACYC-Duet1 plasmid containing the *E. timonensis groEL-ES* genes (Dataset S5). Transformed cells with kanamycin and chloramphenicol resistance were grown in 50 mL LB medium with 50 mg/L kanamycin, 25 mg/L chloramphenicol, and 0.2 mM riboflavin at 37 °C with shaking (180 rpm). When cultures reached an OD_600_ of 0.6, protein expression was induced by addition of isopropylthio-β-galactoside (IPTG) to a final concentration of 0.25 mM. The cultures were then incubated at 15 °C with shaking (180 rpm) for ∼15 h. Cultures aliquots of 15 mL were harvested by centrifugation at 3,220 × *g* for 15 min at 4 °C, and cell pellets were frozen at −80 °C until further use. Cell pellets were transferred and thawed in an anerobic chamber (Coy Laboratory Products) located in a cold room at 4 °C under an atmosphere of ∼3% H_2_ and N_2_ as the balance. The pellets were resuspended in O_2_-free 50 mM potassium phosphate (pH 7.5) buffer, 300 mM NaCl, and 10% glycerol to achieve an OD_600_ = 10. The cells were lysed by sonication for a total of 1 min, with cycles of 2.5 s on and 10 s off. In an anaerobic chamber (Coy Laboratory Products) under an atmosphere of ∼3% H_2_ and N_2_ as the balance, reactions (0.05 mL) containing 0.02 mL of crude lysate, 10 mM acetyl-CoA (CoALA Biosciences, Austin, TX), and 10 mM γbb in O_2_-free 100 mM Tris⋅HCl (pH 8.0) buffer were incubated at room temperature for 1 h and analyzed by LC–MS using the methods for CoA and TMA detection (*SI Appendix*).

### CoA Transferase In Vitro Activity Assay.

Reactions (50 μL) containing 0.01 mM BbuB, 0.01 mM BbuC, 10 mM acetyl-CoA, and 10 mM γbb in 100 mM Tris⋅HCl (pH 8.0) buffer were incubated at room temperature for 1 h and analyzed by LC–MS using the CoA detection method (*SI Appendix*).

### TMA-Lyase In Vitro Activity Assay.

Protein solutions of BbuA, BbuB, and BbuC were deoxygenated on ice by eight rapid cycles of vacuum and N_2_ gas for a total of four times. In an anaerobic chamber (Coy Laboratory Products) under an atmosphere of ∼3% H_2_ and N_2_ as the balance, reactions (0.1 mL) containing 0.2 mM BbuA, 0.01 mM BbuB, 0.01 mM BbuC, 10 mM acetyl-CoA, and 10 mM γbb in O_2_-free 100 mM Tris⋅HCl (pH 8.0) buffer were incubated at room temperature for 1 h and analyzed by LC–MS using the methods for CoA and TMA detection (*SI Appendix*). Reduced BbuA was prepared by preincubation of BbuA with 10 mM sodium dithionite prior to reaction initiation. Reactions with 10 mM γbb-CoA substrate excluded BbuB, BbuC, γbb, and acetyl-CoA.

### LC–MS Sample Preparation and Analytical Methods.

Sample preparation and analytical methods for detection and quantification of γbb, γbb analogs, TMA, butyrate, and CoA-thioester compounds are described in detail in the *SI Appendix*.

### Bioinformatic Analyses.

#### Genome searches.

The *E. timonensis* BbuA protein sequence was used in a Basic Local Alignment Search Tool (BLAST) search of the National Center for Biotechnology Information nonredundant protein database, the UniProt database (release 2019_07), and the Joint Genome Institute-Integrated Microbial Genomes database of all isolates. Hits with >70% amino acid sequence identity were considered BbuA homologs. Searches were also conducted of the HMP1 reference genomes and genomes from the human gut bacteria culture collections described in Dataset S3. No additional hits were identified from these searches. Next, collections of human MAGs (Dataset S3) were performed using a tblastn search and an e-value cutoff of <0.0001. Hits were manually evaluated and further filtered using a >70% amino acid sequence identity cutoff. Contigs containing hits were annotated using the Galaxy webtool Prokka (62, 63).

#### Phylogenetics.

A multiple sequence alignment was generated using MAFFT v7.455 (64) of the BbuA homolog protein sequences and 2,388 representatives of protein clusters with >80% amino acid sequence identity from the top 10,000 hits of a BLAST search of the UniProt database (release 2019_07) using the *E. timonensis* SN18 BbuA protein as a query. A maximum-likelihood phylogenetic tree was constructed using IQ-TREE v1.6.12 (65) with the LG+F0+G12 model and visualized using FigTree v1.4.4 (66). Branch supports were calculated using the aBayes method (67).

#### Metagenome and metatranscriptome searches and quantification.

BbuA homolog protein sequences (Dataset S6) were used to generate a protein database. A negative control protein database was made using protein sequences acquired from the following steps: 1) the top 10,000 results were obtained from a BLAST search of the UniProt database (release 2019_07) using *E. timonensis* SN18 BbuA as the query, excluding BbuA homologs described; 2) these sequences were clustered using UniRef50 (release 2019_07), resulting in 288 representative proteins; and 3) each unique sequence from step 1 between 50% and 70% sequence identity to *E. timonensis* SN18 BbuA was added to the list from step 2. The CntA protein database consisted of *E. coli* YeaW (UniProt P0ABR8), *A. baumannii* CntA (UniProt D0C9N6), and *Klebsiella pneumoniae* CntA (UniProt A0A377WGT7).

Human studies reporting the stool meta’omic data that were analyzed in this work are listed in Dataset S4. A blastx DIAMOND (68) search with an e-value cutoff of <0.0001 and a percent amino acid sequence identity of >50% was performed using the raw shotgun metagenome or metatranscriptome sequencing reads against the BbuA, CntA, and negative control databases. If the highest sequence identity hit for the read to a BbuA protein was greater than or equal to that of a negative control protein and the sequence identity to the BbuA protein was >70%, then that read was considered a positive hit for a *bbuA* gene or transcript. The positive hits for each metagenome sample were summed and then normalized by RPKM and average genome size (AGS) using the following equations:

$$\text{RPKM}=\frac{\left(\frac{\text{reads}}{\text{total reads}}\right)/10^{6}}{\text{gene length}\left[\text{kb}\right]}$$

$$\text{Abundance}=\text{RPKM}\times \text{AGS}\times 10^{-9}.$$

Average genome size for each sample was calculated using MicrobeCensus (69). Plots and statistical analyses were performed using the ggplot2 package v3.3.2 (70) and R v3.6.0 (71).

## Supplementary Material

Supplementary File

Supplementary File

Supplementary File

Supplementary File

Supplementary File

Supplementary File

Supplementary File

## Data Availability

Gene expression profiling by high-throughput sequencing data have been deposited in Gene Expression Omnibus accession no. (GSE165976). All other study data are included in the article and/or supporting information.
