# Supplementary material for: Elucidation of an anaerobic pathway for metabolism of l-carnitine–derived γ-butyrobetaine to trimethylamine in human gut bacteria
Source: Proc Natl Acad Sci U S A. 2021 Aug 6;118(32):e2101498118. doi: 10.1073/pnas.2101498118 (PMC8364193; doi:10.1073/pnas.2101498118)
Supplement: Supplementary File [file pnas.2101498118.sapp.pdf]

PNAS
www.pnas.org

Supplementary Information for

Enzyme discovery defines an anaerobic pathway for metabolism of L-carnitine-derived γ-butyrobetaine to trimethylamine by human gut bacteria

Lauren J. Rajakovich, Beverly Fu, Maud Bollenbach, Emily P. Balskus*

Emily P. Balskus
Email: balskus@chemistry.harvard.edu

This PDF file includes:

Extended Materials and Methods
Tables S1 to S2
Figures S1 to S16
Legends for Datasets S1 to S6

Other supplementary materials for this manuscript include the following:

Datasets S1 to S6

EXTENDED MATERIALS AND METHODS

Cloning and plasmids. Genomic DNA (gDNA) was extracted from *E. timonensis* SN18 cultures using the DNeasy UltraClean Microbial Kit (Qiagen, Germantown, MD) according to the manufacturer's protocol. Oligonucleotide primers were synthesized by Sigma-Aldrich. Genes from the *bbu* gene cluster were amplified from 10 ng *E. timonensis* SN18 gDNA in a 50 μL reaction containing 0.5 μM of forward and reverse primers (**Dataset S5**) and Phusion High-Fidelity PCR Master Mix with HF buffer (New England Biolabs, Ipswich, MA). A plasmid based on the pET28a(+) vector for constitutive expression was constructed using the following protocol and verified by Sanger sequencing. The empty vector pET28a(+) was digested with the restriction enzymes FspI and BamHI (New England Biolabs, Ipswich, MA). After PCR cleanup using the Zymo DNA Clean and Concentrator Kit (Zymo, Irvine, CA), the linearized vector was assembled with a GBlock (**Dataset S5**) carrying the proD promoter (1) using HiFi DNA Assembly Master Mix (New England Biolabs, Ipswich, MA). Vectors used to construct plasmids containing the *bbu* genes were linearized by PCR amplification from 10 ng vector template DNA in a 50 μL reaction containing 0.5 μM of forward and reverse primers (**Dataset S5**) and Phusion High-Fidelity PCR Master Mix with HF buffer (New England Biolabs, Ipswich, MA). Thermocycling was carried out in a Bio-Rad C100 thermal cycler using the following parameters: denaturation at 95 °C for 2 min, followed by 35 cycles of 95 °C for 30 s, 60 °C for 30 s, 72 °C for 1 min/kb, and a final extension at 72 °C for 5 min. PCR reactions were analyzed by agarose gel electrophoresis with ethidium bromide staining and purified using the Illustra GFX PCR DNA and Gel Band Purification kit (GE Healthcare, Chicago, IL). Reactions (10 μL) containing 50 ng linearized vector, 4 molar equivalents of gene insert, and Gibson Assembly Master Mix (New England Biolabs, Ipswich, MA) were incubated at 50 °C for 1 h and

transformed into *E. coli* TOP10 chemically competent cells (Thermo Fischer Scientific, Waltham, MA). Transformants were screened by antibiotic selection. Plasmids were purified using the E.Z.N.A. Plasmid Mini Kit I (Omega Bio-tek, Norcross, GA) according to the manufacturer's protocol and confirmed by Sanger sequencing by Eton Bioscience (Charlestown, MA).

RNA sample preparation, sequencing, and data analysis. Total RNA was isolated by bead beating using 0.1 mm dia. Zirconia/Silica Beads (BioSpec Products, Bartlesville, OK) at 10 m/s for 90 s to lyse cells and then using the Zymo Research Direct-Zol RNA MiniPrep Plus kit (Zymo, Irvine, CA) according to the manufacturer's protocol. Illumina cDNA libraries were generated using a modified version of the RNAtag-Seq protocol (2). Briefly, 500 ng of total RNA was fragmented, depleted of genomic DNA, and dephosphorylated prior to its ligation to DNA adapters carrying 5′-AN8-3′ barcodes with a 5′ phosphate and a 3′ blocking group. Barcoded RNAs were pooled and depleted of rRNA using the RiboZero rRNA depletion kit (Epicentre, Madison, WI). These pools of barcoded RNAs were converted to Illumina cDNA libraries in three main steps: (i) reverse transcription of the RNA using a primer designed to the constant region of the barcoded adaptor; (ii) addition of a second adapter on the 3' end of the cDNA during reverse transcription using SmartScribe RT (Clontech Biotechnologies, Mountain View, CA) as previously described (2); (iii) PCR amplification using primers that target the constant regions of the 3' and 5' ligated adaptors and contain the full sequence of the Illumina sequencing adaptors. cDNA libraries were sequenced on Illumina HiSeq 2500.

For the analysis of RNAtag-Seq data, reads from each sample in the pool were identified based on their associated barcode using custom scripts, and up to one mismatch in the barcode was allowed with the caveat that it did not enable assignment to more than one barcode. Barcode

sequences were removed from the first read as were terminal G's from the second read that may have been added by SMARTScribe during template switching. Reads were aligned to the *E. timonensis* SN18 genome (NCBI GenBank: FLKM00000000.1) using BWA (3) and read counts were assigned to genes and other genomic features using custom scripts. Differential expression analysis was conducted with DESeq2 (4).

Heterologous Expression in *E. coli* and protein purification. Plasmids (**Dataset S5**) containing the *E. timonensis bbuB* gene or the *bbuC* gene in a pET28a(+) vector or the *Sinorhizobium meloloti bcoAB* gene in a pET22 vector (5) were used to transform *E. coli* BL21 Codon-Plus (DE3-*pLys*) chemically competent cells (Invitrogen, Carlsbad, CA). Transformed cells with kanamycin and chloramphenicol resistance were cultured at 37 °C with shaking (180 rpm) in rich Luria Broth (LB) medium with 50 mg/L kanamycin and 25 mg/L chloramphenicol. When cultures reached an OD_{600} between 0.6-0.8, protein expression was induced by addition of IPTG to a final concentration of 0.25 mM. The cultures were then incubated at 15 °C with shaking (180 rpm) for ~18 h and harvested by centrifugation at 6,000 × *g* for 15 min at 4 °C. Cell pellets were flash frozen in liquid N_2 and stored at –80 °C until further use. Cells were resuspended in lysis buffer [50 mM *tris*-(hydroxymethyl)aminomethane (Tris)-HCl (pH 7.5) buffer, 150 mM NaCl, and 10% glycerol] at a ratio of approximately 10 mL buffer per 1 g of wet cell mass. The suspension was lysed by passaging twice through a cell disrupter at 12,000 psi and centrifuged at 20,000 × *g* for 30 min at 4 °C. The supernatant was loaded onto a Ni^{2+}-NTA immobilized affinity chromatography column (~10 mL resin per 100 mL lysate) pre-equilibrated with lysis buffer. After loading, 3 column volumes of lysis buffer were passed through the column, followed by 5 column volumes of wash buffer (lysis buffer with 50 mM imidazole). Protein elution was achieved by passing elution buffer (lysis buffer with 250 mM imidazole)

over the column. Fractions containing the protein of interest were combined and concentrated at 3,500 × *g* using a 10K MWCO Amicon Ultra-15 Centrifugal Filter Unit (Millipore Sigma, Burlington, MA). The concentrated protein was then dialyzed three times for 4 h each against 100 equivalent volumes of lysis buffer. The protein was frozen in liquid N_2 and stored at –80 °C. Protein purity was assessed by SDS-PAGE with Coomassie staining (**Figure S3**), and protein concentration was determined by using the molar absorption coefficient at 280 nm.

E. coli BL21 competent cells (Invitrogen, Carlsbad, CA) were co-transformed with a pET28a plasmid containing the *E. timonensis bbuA* gene and a pACYC-Duet1 plasmid containing the *E. timonensis groEL-ES* genes (**Dataset S5**). Transformed cells with kanamycin and chloramphenicol resistance were cultured at 37 °C with shaking (180 rpm) in LB medium with 50 mg/L kanamycin, 25 mg/L chloramphenicol, and 0.2 mM riboflavin. When cultures reached an OD_{600} of 0.6, protein expression was induced by addition of IPTG to a final concentration of 0.25 mM. The cultures were then incubated at 15 °C with shaking (180 rpm) for ~18 h and harvested by centrifugation at 6,000 × *g* for 15 min at 4 °C. Cell pellets were flash frozen in liquid N_2 and stored at –80 °C until further use. Cells were resuspended in lysis buffer [50 mM potassium phosphate (pH 7.5) buffer, 300 mM NaCl, 10% glycerol, and 1 mM FAD] at a ratio of approximately 10 mL buffer per 1 g of wet cell mass. The suspension was lysed by passaging twice through a cell disrupter at 12,000 psi and centrifuged at 20,000 × *g* for 30 min at 4 °C. The supernatant was loaded onto a Ni^{2+}-NTA immobilized affinity chromatography column (~10 mL resin per 100 mL lysate) pre-equilibrated with lysis buffer without FAD. After loading the lysate, 3 column volumes of lysis buffer without FAD were passed through the column, followed by 3 column volumes of wash buffer A [50 mM potassium phosphate (pH 7.5) buffer, 300 mM NaCl, 10% glycerol, and 50 mM imidazole] and 3 column volumes of wash buffer B [50 mM

potassium phosphate (pH 7.5) buffer, 300 mM NaCl, 10% glycerol, and 75 mM imidazole]. Protein elution was achieved by passing elution buffer [50 mM potassium phosphate (pH 7.5) buffer, 300 mM NaCl, 10% glycerol, and 250 mM imidazole] over the column. Fractions containing the protein of interest were combined and immediately concentrated at 3,500 × *g* using a 30K MWCO Amicon Ultra-15 Centrifugal Filter Unit (Millipore Sigma, Burlington, MA). The protein was frozen in liquid N_2 without buffer exchange and stored at –80 °C. Protein purity was assessed by SDS-PAGE with Coomassie staining (**Figure S3**), and protein concentration was determined by using the molar absorption coefficient at 280 nm. FAD-bound protein concentration was determined by using a molar absorption coefficient of 11,300 $M^{-1}cm^{-1}$ at 450 nm.

Affinity pull-down chromatography of the BbuB-Strep protein. A plasmid (**Dataset S5**) containing the *E. timonensis bbuB* gene in a pPR-IBA1 vector was used to transform *E. coli* BL21 Codon-Plus (DE3-*pLys*) chemically competent cells (Invitrogen, Carlsbad, CA). Transformed cells with ampicillin and chloramphenicol resistance were cultured at 37 °C with shaking (180 rpm) in 2 L of rich Luria Broth (LB) medium with 50 mg/L ampicillin and 25 mg/L chloramphenicol. When cultures reached an OD_{600} between 0.6-0.8, protein expression was induced by addition of IPTG to a final concentration of 0.25 mM. The cultures were then incubated at 15 °C with shaking (180 rpm) for ~18 h and harvested by centrifugation at 6,000 × *g* for 15 min at 4 °C. Cell pellets were flash frozen in liquid N_2 and stored at –80 °C until further use. Cells were resuspended in 30 mL lysis buffer [50 mM *tris*-(hydroxymethyl)aminomethane (Tris)-HCl (pH 7.5) buffer, 150 mM NaCl, and 10% glycerol]. The suspension was lysed on ice by sonication at 25% amplitude for a total of 3 min with 10 s off every 2.5 s. The crude lysate was combined in equal volume with crude lysate of BbuC that had been heterologously

expressed as described in the previous section. The crude lysate mixture was centrifuged at 15,000 × *g* for 45 min and then the supernatant was loaded onto a Ni^{2+}-NTA immobilized affinity chromatography column (5 mL resin) pre-equilibrated with lysis buffer. Next, the column was washed with 20 mL of lysis buffer with 25 mM imidazole, followed by 20mL column volumes of lysis buffer with 50 mM imidazole, 15 mL of lysis buffer with 100 mM imidazole, and 15 mL of lysis buffer with 250 mM imidazole. Samples of each wash fraction were analyzed by (1) SDS-PAGE with Coomassie staining and (2) Western blot with 1:25,000 dilution of Strep-Tactin HRP-conjugate (IBA LifeSciences, Göttingen, Germany) and staining using the Pierce CN/DAB Substrate Kit (Thermo Scientific, Waltham, MA).

Liquid chromatography – mass spectrometry sample preparation and analytical methods

γbb and TMA analysis. Samples were diluted 1:10 (*v/v*) in solvent A composed of 95:5 acetonitrile (ACN):100 mM ammonium formate (MS grade) in H_2O with 0.02% formic acid (MS grade) and centrifuged at 3,220 × *g* for 10 min. The extracted supernatant samples were further diluted 1:4 (*v/v*) in solvent A and kept at 4 °C until analysis (performed within 24 h).

Liquid chromatography was conducted using an Agilent 1200 Series G1312B Binary Pump (Agilent Technologies, Santa Clara, CA) instrument and a Phenomenex HILIC column (30 mm × 2.1 mm, 2.5 μm). Samples (2 μL) were injected onto the column equilibrated with 100% solvent A and the following gradient method was applied over 7.5 min with a flow rate of 0.6 mL/min: 1) Solvent A was maintained at 100% from 0 to 1 min, 2) solvent B was increased to 90% from 1 to 3 min, 3) solvent B was maintained at 90% from 3 to 4.5 min, 4) solvent B was decreased to 0% from 4.5 to 6 min, and 5) the column was re-equilibrated with 100% solvent A from 6 to 7.5 min. Tandem MS/MS detection was performed with an Agilent 6410 Triple Quadrupole (Agilent Technologies, Santa Clara, CA) instrument with electron spray ionization

in positive mode (ESI+). The source parameters were gas temperature of 200 °C, gas flow of 10 L/min, nebulizer pressure of 45 psi, and capillary voltage of 4000 V. Conditions for MS/MS fragmentation for the metabolites of interest are found in **Table S1**. Standards were prepared according to the same methods used for experimental samples and standard curves were used to quantify metabolite concentrations.

Table S1. MS/MS parameters used to detect metabolites of interest.

Metabolite	Parent ion $[M+H]^+$ (*m/z*)	Daughter ion $[M+H]^+$ (*m/z*)	Dwell (ms)	Fragmentor (V)	CE (V)
**γbb**	146	87	200	110	21
**TMA**	60	45	200	110	26
**DL-Carnitine**	162	103	200	110	21
**GABA**	104	87	200	110	21

Derivatization of fatty acids with 2-nitrophenylhydrazine/1-ethyl-3-(3-dimethylaminopropyl)-carbodiimide and analysis. Samples were diluted 1:10 (*v/v*) in 95:5 ACN:100 mM ammonium formate (MS grade) in H_2O with 0.02% formic acid (MS grade) and centrifuged at 3,220 × *g* for 10 min. The extracted supernatant samples (10 µL) were diluted 1:10 (*v/v*) in a solution of 10% 1:1 pyridine:HCl (pH 3.5-5) in H_2O. A pre-mixed derivatization solution consisting of equal parts 0.29 M 1-ethyl-3-(3-dimethylaminopropyl)-carbodiimide (EDC) hydrochloride (dissolved in water) and 0.12 M 2-nitrophenylhydrazine (2-NPH; dissolved in 0.25 M HCl) was prepared fresh and 20 µL of this solution was added to 100 µL of 100-fold diluted sample. The derivatization reactions were heated at 60 °C for >15 min and then diluted 1:10 (*v/v*) in H_2O prior to LC–MS analysis.

Liquid chromatography was conducted using an Agilent 1200 Series G1312B Binary Pump (Agilent Technologies, Santa Clara, CA) instrument and an Agilent Extend C18 column (50 mm × 2.1 mm, 1.8 µm). The derivatized samples (2 µL) were injected onto the column equilibrated with 95% H_2O (solvent A) and 5% ACN (solvent B). The following gradient method was applied

over 13 min with a flow rate of 0.3 mL/min: 1) solvent A was maintained at 95% from 0 to 4 min, 2) solvent B was increased to 100% from 0 to 4 min, 3) solvent B was maintained at 100% from 4 to 7 min, 4) solvent B was decreased to 5% from 7 to 9 min, and 5) the column was re-equilibrated with 95% solvent A from 9 to 13 min. An MS scan was performed with an Agilent 6410 Triple Quadrupole (Agilent Technologies, Santa Clara, CA) instrument with electron spray ionization in negative mode (ESI–). The source parameters were a gas temperature of 325 °C, a gas flow of 12 L/min, a nebulizer pressure of 40 psi, and a capillary voltage of 4000 V. The detection mode was set to a scan range of 100–400 *m/z* and a scan time of 500 ms with a fragmentor voltage of 135 V and a step size of 0.1 amu. The parent $[M\text{-}H]^-$ ions for derivatized short-chain fatty acids were monitored, including formate (180 *m/z*), acetate (194 *m/z*), propionate (208 *m/z*), and butyrate (222 *m/z*). Butyrate concentrations were determined by comparison to a 10 µM sodium $^{13}C_4$-butyrate internal standard added to the derivatization solution.

CoA-thioester analysis. Cell cultures (5 mL) or whole cell suspensions (1.5 mL) were centrifuged at 3,220 × *g* or 16,000 × *g*, respectively, for 10 min at 4 °C. Cell pellets were resuspended in 0.2 mL of cold solution of 40:20:20 ACN:MeOH:H_2O. Resuspended cells were incubated at –20 °C for 30 min and then centrifuged at 16,000 × *g* for 30 min. The supernatant was transferred to a new tube and dried using a Genevac EZ-2.3 Elite Evaporation System (Genevac Ltd, Ipswich, UK) for 1 h. The dried samples were resuspended in 80 µL H_2O for LC–MS analysis. Samples (20 µL) from lysate or *in vitro* reactions were quenched in 2 µL concentrated acetic acid, diluted 1:10 in H_2O, and incubated at –20 °C for 30 min before centrifugation at 16,000 × *g* for 10 min. Samples were further diluted 1:4 in H_2O and kept at 4

°C prior to LC–MS analysis. Standards were prepared according to the same methods used for experimental samples.

Liquid chromatography was conducted using a Waters Acquity UPLC H-Class System (Waters Corporation, Milford, MA) instrument and a Waters Acquity UPLC BEH C18 column (2.1 × 50 mm, 1.7 µm). The samples (2 µL) were injected onto the column equilibrated with 100% 30 mM ammonium acetate (solvent A). The following gradient method was applied over 5 min with a flow rate of 0.8 mL/min: 1) solvent A was maintained at 100% from 0 to 0.5 min, 2) solvent B (acetonitrile + 0.1 % formic acid) was increased to 20% from 0.5 to 4 min, 3) solvent B was increased to 100% from 4.0 to 4.1 min, 4) solvent B was maintained at 100% from 4.1 to 4.4 min, 5) solvent B was decreased to 0% from 4.4 to 4.5 min, and 5) the column was re-equilibrated with 100% solvent A from 4.5 to 5 min. MS detection was performed with a Waters Xevo TQ-S (Waters Corporation, Milford, MA) instrument with electron spray ionization in positive mode (ESI+). The source parameters were gas temperature of 500 °C, desolvation gas flow of 1000 L/h, cone gas flow of 150 L/h nebulizer pressure of 7 bar, capillary voltage of 0.5 kV and cone voltage of 3 V. Conditions for tandem MS/MS detection were optimized using an acetyl-CoA standard. Conditions for a parent MS/MS scan of the 136 *m/z* mass fragment (corresponding to adenine loss) were a scan range of 800–910 *m/z*, scan time of 0.15 s, collision energy of 53 V, and cone voltage of 70 V. Conditions for an MS scan were a scan range of 800–910 *m/z*, cone voltage of 70 V, and scan time of 0.15 s. Conditions for MS/MS fragmentation for the metabolites of interest are found in **Table S2**.

Table S2. MS/MS parameters used to detect metabolites of interest.

Metabolite	Parent ion $[M+H]^+$ (*m/z*)	Daughter ion $[M+H]^+$ (*m/z*)	Dwell (ms)	Fragmentor (V)	CE (V)
**Acetyl-CoA**	810.1957	135.9617	52	70	54
**Crotonyl-CoA**	836.5	135.9617	52	70	54
**Butyryl-CoA**	838.12	135.9617	52	70	54

**3-Hydroxybutyryl-CoA**	854.0	135.9617	52	70	54
**γbb-CoA**	895.5	135.9617	52	70	54

Chemical synthesis of crotonyl-CoA. Crotonyl-CoA was synthesized according to a published protocol (6) with the following modifications. One molar equivalent of solid Coenzyme A trilithium salt (CoAla Biosciences; 35 mg, 0.046 mmol) was dissolved in 3 mL of ice-cold water. To this solution, 1.5 molar equivalents of crotonic anhydride (Sigma Aldrich; 10 μL, 0.068 mmol) was added and the pH was adjusted to pH 7.0 using a saturated solution of $NaHCO_3$. The reaction was stirred in an ice bath for 1.5 h. Reaction progress was monitored using the DTNB "Ellman's" assay to detect free CoA. The reaction was quenched by adding 48 μL formic acid to lower the pH below 3 and was kept frozen at –20 °C prior to purification. The crotonyl-CoA product was purified using an Thermo Scientific Dionex UltiMate 3000 HPLC instrument and a Thermo Scientific Hypersil GOLD aQ C18 preparative column (250 × 20 mm, 5 μM). The reaction solution was injected onto the column equilibrated with 100% solvent A (10 mM ammonium acetate) and the following gradient method was applied over 21 min with a flow rate of 8 mL/min: 1) solvent B was increased to 40% from 0 to 14 min, 2) solvent B was increased to 95% from 14 to 15 min, 3) solvent B was maintained at 95% from 15 to 16 min, 4) solvent B was decreased to 0% from 16 to 16.5 min, and 5) the column was re-equilibrated with 100% solvent A from 16 to 21 min for subsequent injections. Fractions with absorption at 260 nm were collected and lyophilized to afford crotonyl-CoA as a white solid (10 mg, 0.012 mmol, **26%**). ^{1}H NMR (400 MHz, D_2O) δ ppm 0.78 (s, 3H), 0.91 (s 3H), 1.87 (d, 3H, J = 6.7 Hz), 2.44 (t, 2H, J = 6.1 Hz), 3.03 (t, 2H, J = 6.1 Hz), 3.35 (t, 2H, J = 6.2 Hz), 3.46 (t, 2H, J = 6.2 Hz), 3.58 (dd, 1H, J = 4.3 Hz, J = 9.3 Hz), 3.85 (dd, 1H, J = 4.3 Hz, J = 9.6 Hz), 4.04 (s, 1H), 4.26 (br s, 2H), 4.61 (br s, 1H), 4.84-4.87 (m, 2H), 6.17-621 (m, 2H), 6.95 (sextet, 1H, J = 7.2 Hz), 8.27 (s, 1H), 8.56 (s, 1H).

1. J. H. Davis, A. J. Rubin, R. T. Sauer, Design, construction and characterization of a set of insulated bacterial promoters. *Nucleic acids research* **39**, 1131-1141 (2011).
2. A. A. Shishkin, *et al.*, Simultaneous generation of many RNA-seq libraries in a single reaction. *Nature methods* **12**, 323-325 (2015).
3. H. Li & R. Durbin, Fast and accurate short read alignment with Burrows–Wheeler transform. *Bioinformatics* **25**, 1754-1760 (2009).
4. M. I. Love, W. Huber, S. Anders, Moderated estimation of fold change and dispersion for RNA-seq data with DESeq2. *Genome Biol* **15**, 550 (2014).
5. P. Bazire, *et al.*, Characterization of L-carnitine metabolism in *Sinorhizobium meliloti*. *Journal of bacteriology* **201**, e00772-00718 (2019).
6. E. J. Simon & D. Shemin, The Preparation of *S*-Succinyl Coenzyme A. *J Am Chem Soc* **75**, 2520-2520 (1953).

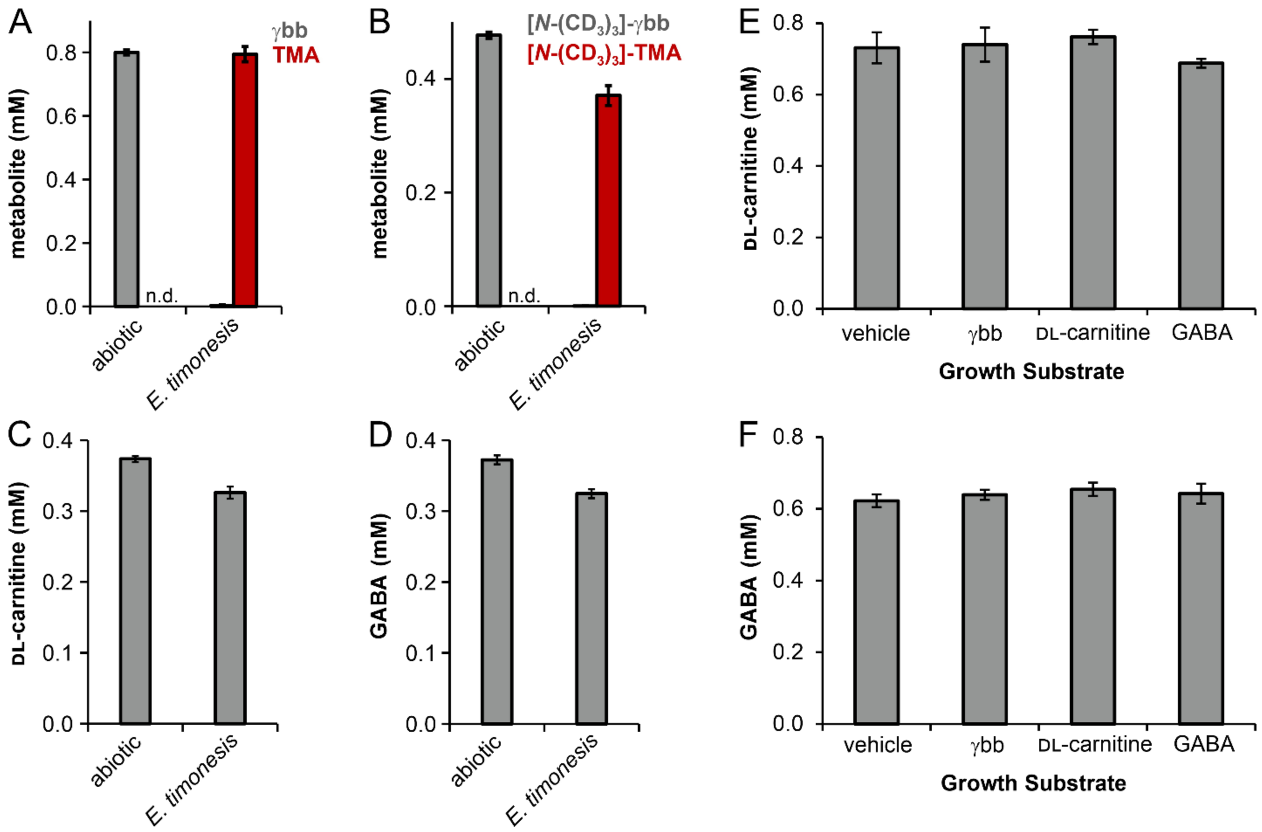

Figure S1. Metabolism of γbb and structural analogs in growing cultures and resting cell suspensions of *E. timonensis*. **(A-D)** Bar plots of metabolite concentrations detected by LC-MS from *E. timonensis* SN18 culture extracts after 20 h of growth. Media were supplemented with **(A)** 0.8 mM γbb, **(B)** 0.4 mM [*N*-(CD_3)$_3$]-γbb, **(C)** 0.4 mM DL-carnitine, or **(D)** 0.4 mM GABA. **(E-F)** Concentration of metabolites detected by LC-MS from extracts of resting cell suspensions of *E. timonensis* SN18 that were grown in the presence of 1 mM γbb, DL-carnitine, GABA, or 1× PBS and incubated with **(E)** 0.8 mM DL-carnitine, or **(F)** 0.8 mM GABA. Error bars represent standard deviation from the mean of three biological replicates.

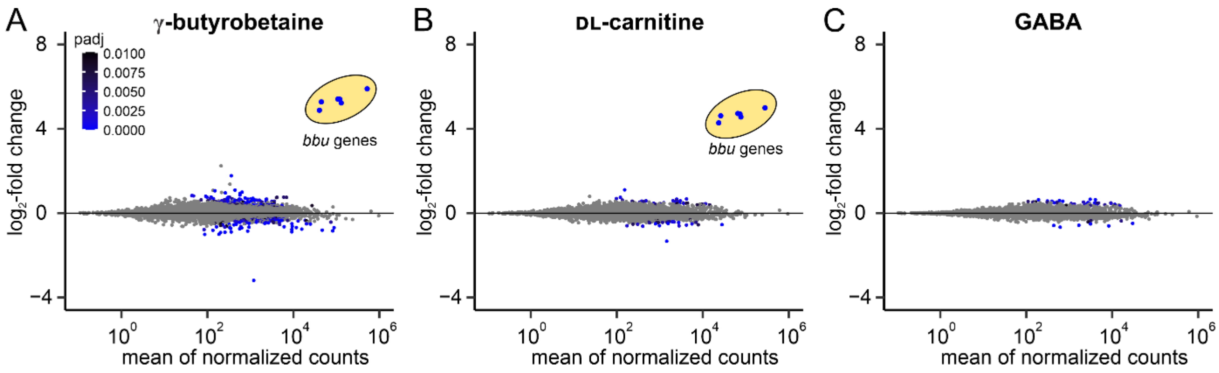

Figure S2. Differential gene expression from *E. timonesis* SN18 cultures supplemented with (**A**) γbb, (**B**) DL-carnitine, or (**C**) GABA at an OD = 0.5 compared to cultures treated with a vehicle* plotted against the mean of normalized counts from three biological replicates of the substrate-induced samples. The *bbu* genes are circled in yellow. Genes with an adjusted *p*-value >0.01 (Wald test) comparing γbb- and vehicle-treated cells are represented by grey circles.

*This set of triplicate samples for each condition were conducted in independent experiments from the data presented in Figure 2 of the main text.

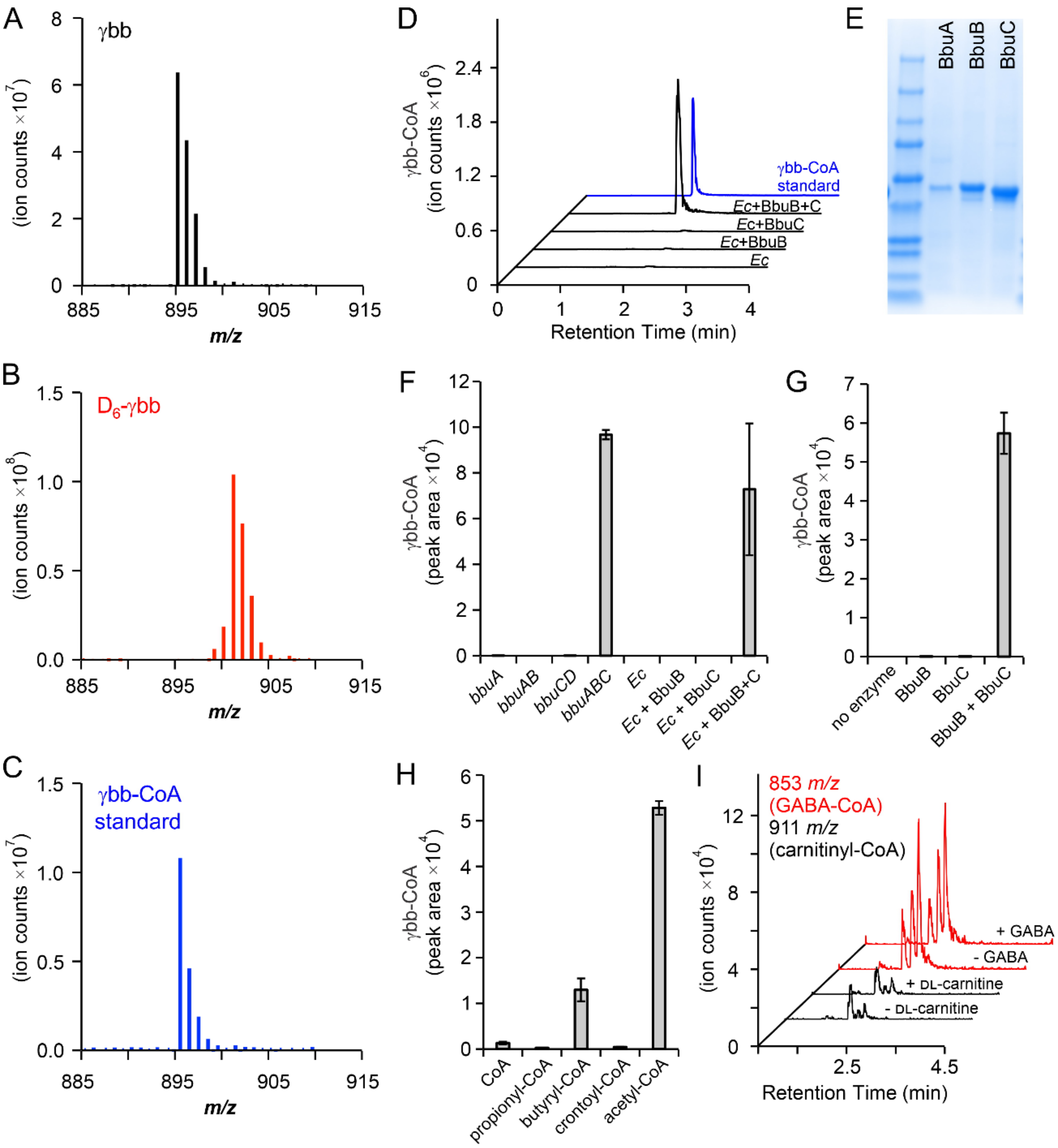

Figure S3. Production of γbb-CoA *in vivo* and *in vitro*. Mass spectra of γbb-CoA from extracts of *E. timonensis* cell suspensions incubated for 40 min with (**A**) γbb or (**B**) D_6-γbb compared to (**C**) a γbb-CoA standard. (**D**) LC–MS/MS selected ion chromatograms of the 136 *m/z* fragment ion of γbb-CoA from 1 h incubations of γbb, acetyl-CoA, and purified BbuB and BbuC with crude lysate of *E. coli* transformed with an empty vector. (**E**) SDS-PAGE Coomassie stained gel of recombinant, purified BbuA, BbuB, and BbuC proteins. (**F-G**) Relative amounts of γbb-CoA determined by LC–MS peak area from (**F**) crude lysate and (**G**) *in vitro* assays. (**H**) Relative amounts of γbb-CoA determined by LC–MS peak area using various short-chain fatty acid CoA donor substrates. (**I**) LC–MS/MS selected ion chromatograms of the 136 *m/z* fragment-parent ion pairs for DL-carnitinyl-CoA and GABA-CoA from 3-h incubations of DL-carnitine or GABA and acetyl-CoA with purified BbuB and BbuC. Error bars shown in panels E–G represent standard deviation from the mean of three biological replicates.

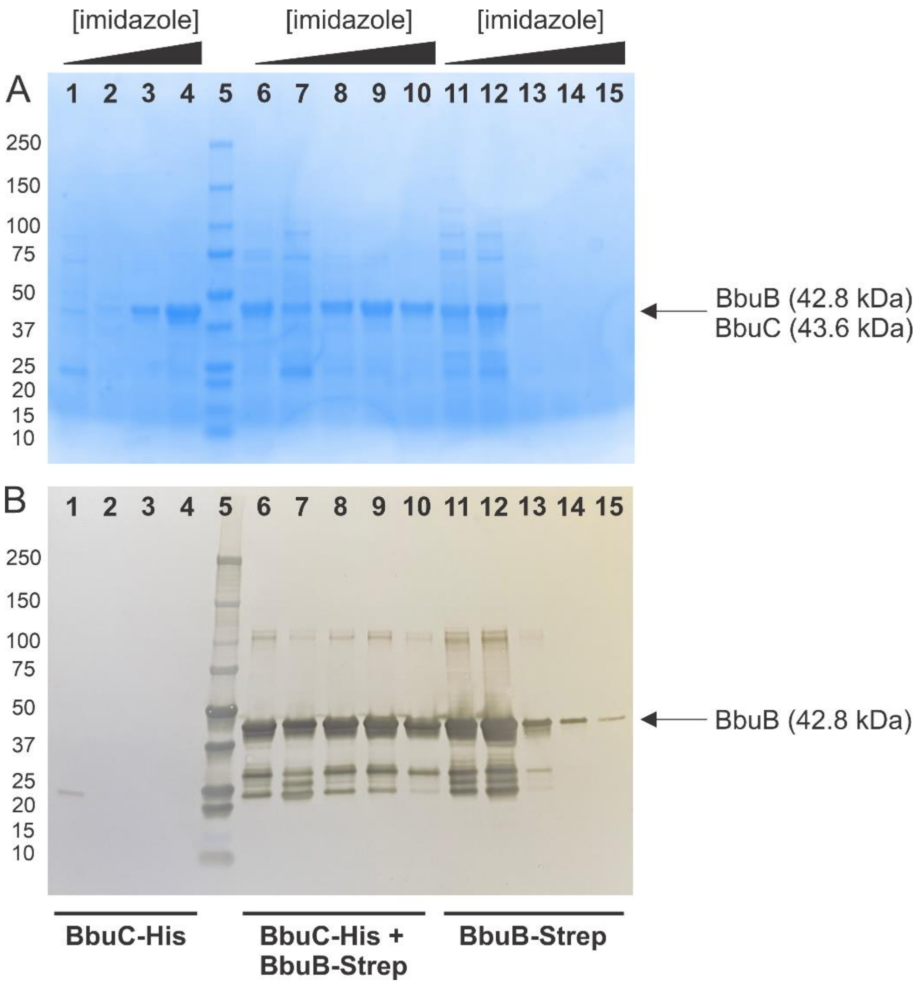

Figure S4. Ni^{2+}-NTA affinity pull-down chromatography of BbuB-Strep with BbuC-His. (**A**) SDS-PAGE Coomassie stained gel image of fractions collected from Ni^{2+}-NTA purification of BbuC-His alone (lanes 1-4: [**lane 1** – 25 mM imidazole, **lane 2** – 50 mM imidazole, **lane 3** – 100 mM imidazole, **lane 4** – 250 mM imidazole]), BbuB-Strep alone (lanes 11-15: [**lane 11** – 15 mM imidazole, **lane 12** – 25 mM imidazole, **lane 13** – 50 mM imidazole, **lane 14** – 100 mM imidazole, **lane 15** – 250 mM imidazole]), and a 1:1 mixture of BbuC-His and BbuB-Strep (lanes 6-10: [**lane 6** – 15 mM imidazole, **lane 7** – 25 mM imidazole, **lane 8** – 50 mM imidazole, **lane 9** – 100 mM imidazole, **lane 10** – 250 mM imidazole]). (**B**) Corresponding western blot of identical samples labeled with a streptavidin-tactin HRP-conjugate antibody to track the BbuB-Strep protein.

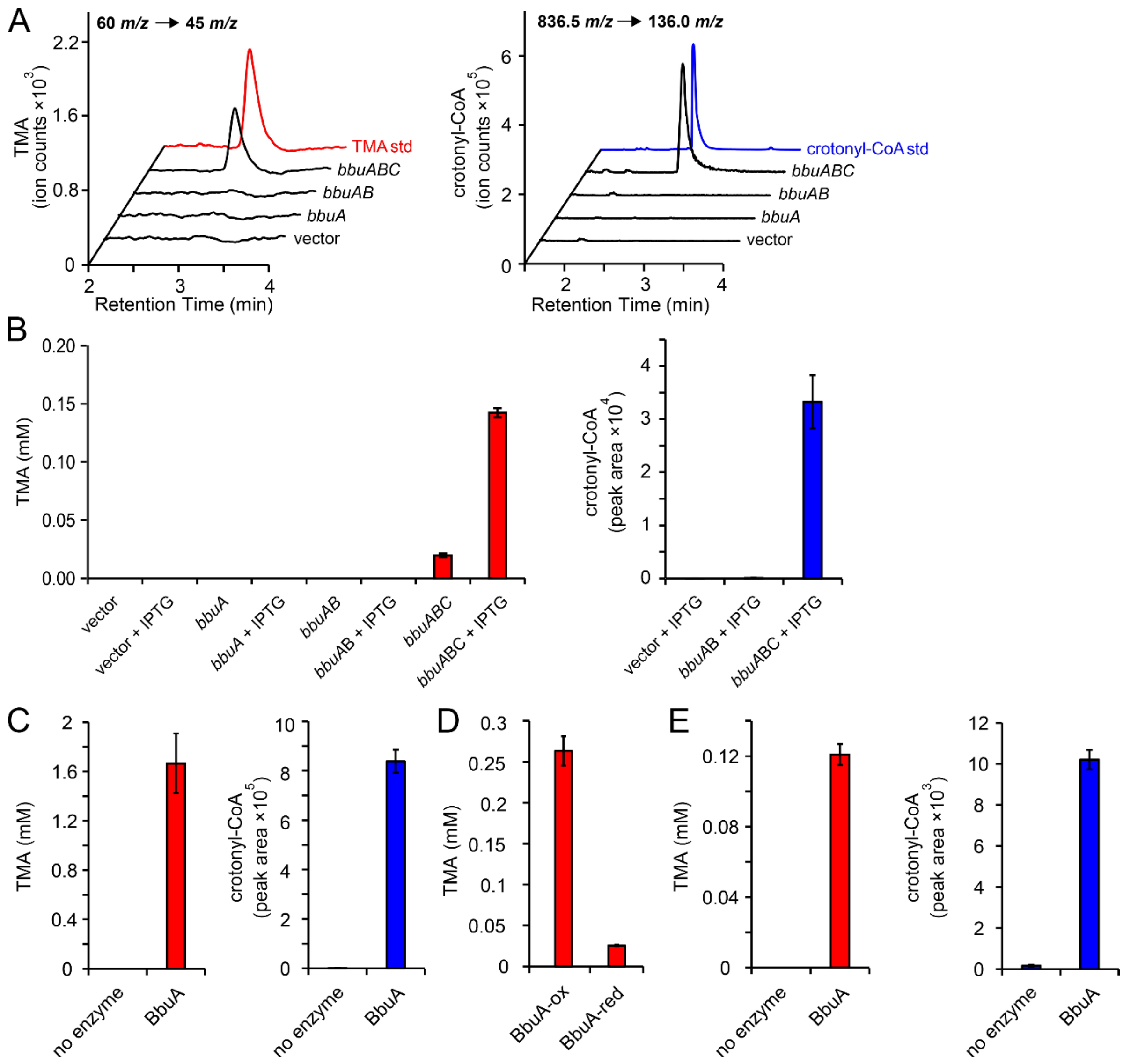

Figure S5. Production of TMA and crotonyl-CoA from *E. coli* gain-of-function experiments and *in vitro* activity assays. (**A**) LC–MS/MS selected ion chromatograms (SIC) of the 136.0 *m/z* fragment-precursor ion pairs of TMA and crotonyl-CoA that were produced from 1 h incubations of γbb and acetyl-CoA with crude lysate of IPTG-induced *E. coli* expressing *bbu* genes or empty vector. (**B**) TMA concentrations and crotonyl-CoA relative amounts determined by LC–MS/MS that were produced from 1 h incubations of γbb and acetyl-CoA with crude lysate of IPTG-induced *E. coli* expressing *bbu* genes or empty vector. (**C**) TMA concentrations and crotonyl-CoA relative amounts determined by LC–MS/MS that were produced from 1 h reactions containing γbb, acetyl-CoA, BbuB, BbuC, and FAD with or without addition of BbuA. (**D**) TMA concentrations determined by LC–MS/MS that were produced from 1 h reactions containing 2 mM γbb, 2 mM acetyl-CoA, 0.01 mM BbuB, 0.01 mM BbuC, and 0.01 mM BbuA (BbuA-ox) or 0.01 mM BbuA pre-treated with 10 mM sodium dithionite (BbuA-red). (**E**) TMA concentrations and crotonyl-CoA relative amounts determined by LC–MS/MS that were produced from 1 h reactions containing γbb-CoA and FAD with or without addition of BbuA.

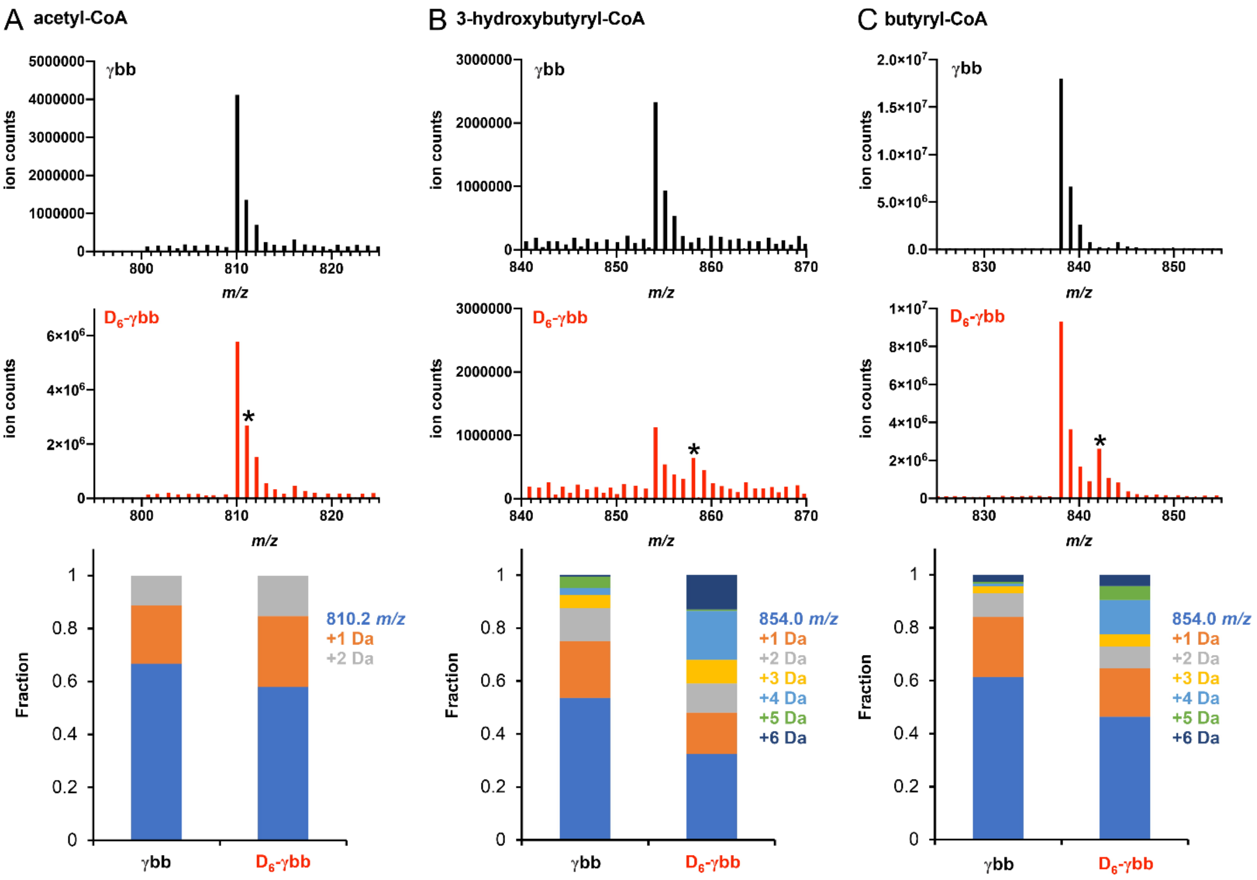

Figure S6. Mass spectra of the products of crotonyl-CoA metabolism [(**A**) acetyl-CoA, (**B**) 3-hydroxybutyryl-CoA, (**C**) butyryl-CoA] from extracts of *E. timonensis* SN18 cell suspensions incubated for 40 min with γbb (black) or D_6-γbb (red). Asterisks indicate increased abundance of ions attributed to deuterium-labeled isotopologs. Stacked bar plots show fraction of metabolite isotopologs.

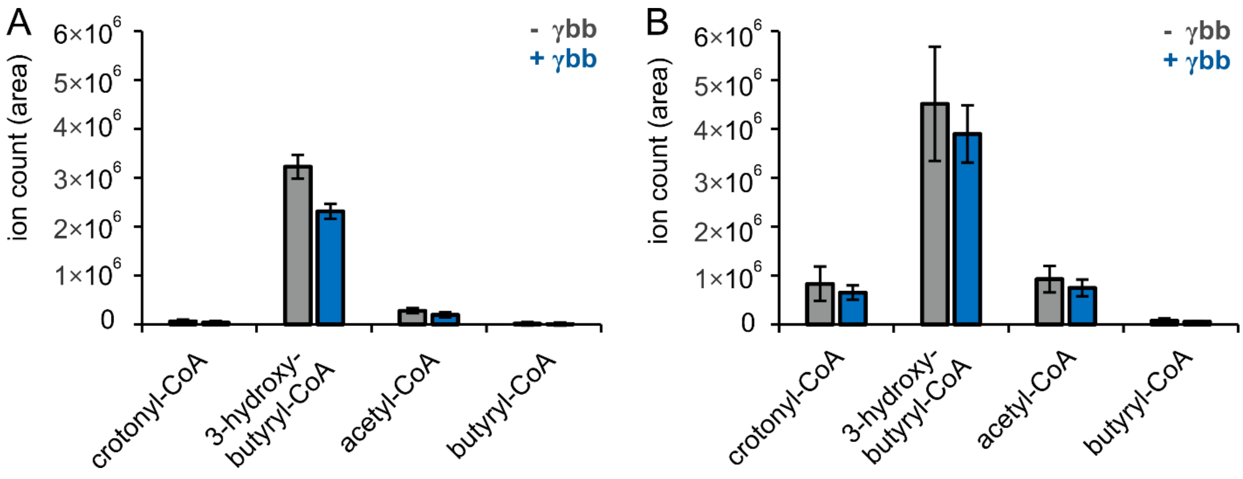

Figure S7. Metabolites detected by LC–MS from 1 h (**A**) and 4 h (**B**) incubations of crotonyl-CoA with crude lysate of *E. timonensis* SN18 that was cultured in the presence (blue) or absence (grey) of γbb. Error bars represent the standard deviation from the mean of three biological replicates.

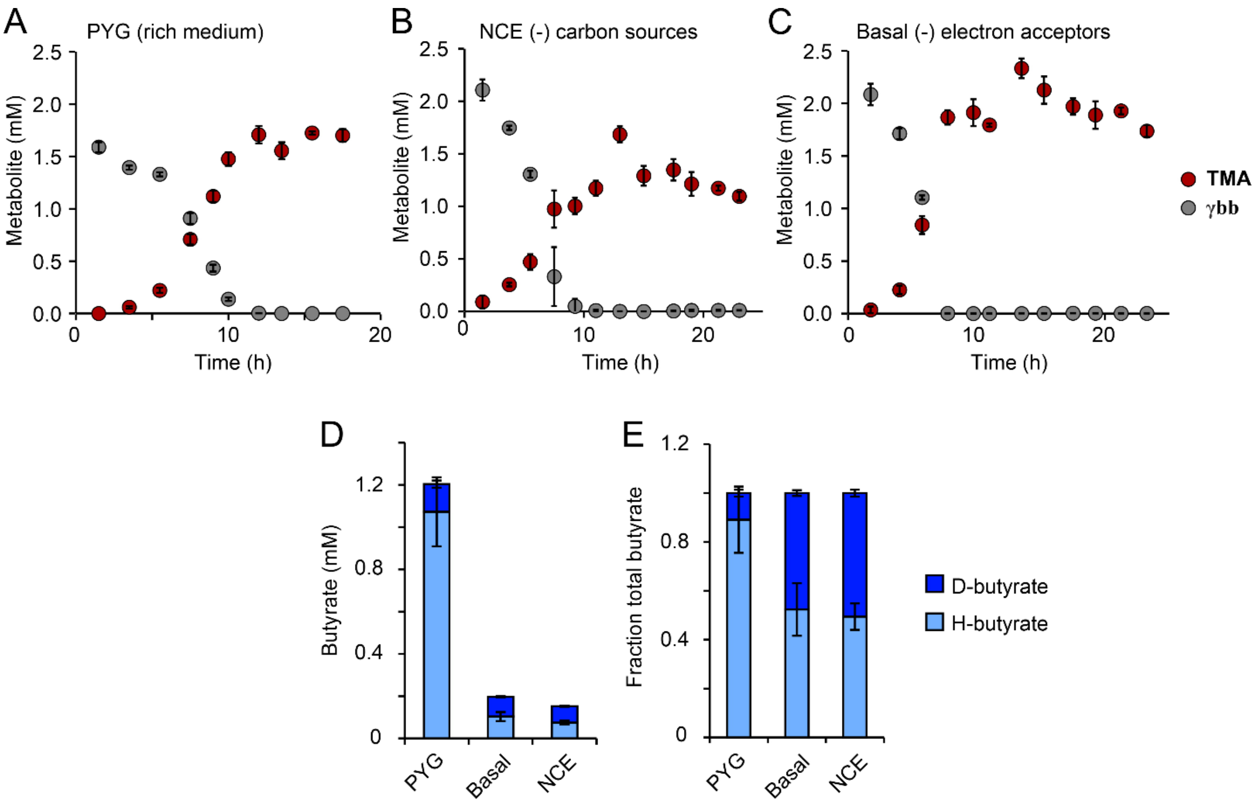

Figure S8. Metabolite consumption and production, monitored by LC–MS, during anaerobic growth of *E. timonensis* SN18 in rich medium (**A**) or minimal media (**B** and **C**) when supplemented with 2 mM γbb. (**D**) Quantification of total butyrate produced during growth in each medium when supplemented with 2 mM D_6-γbb. (**E**) Fraction of deuterium-labeled butyrate produced during growth in each medium when supplemented with D_6-γbb. Error bars represent standard deviation from the mean of three biological replicates.

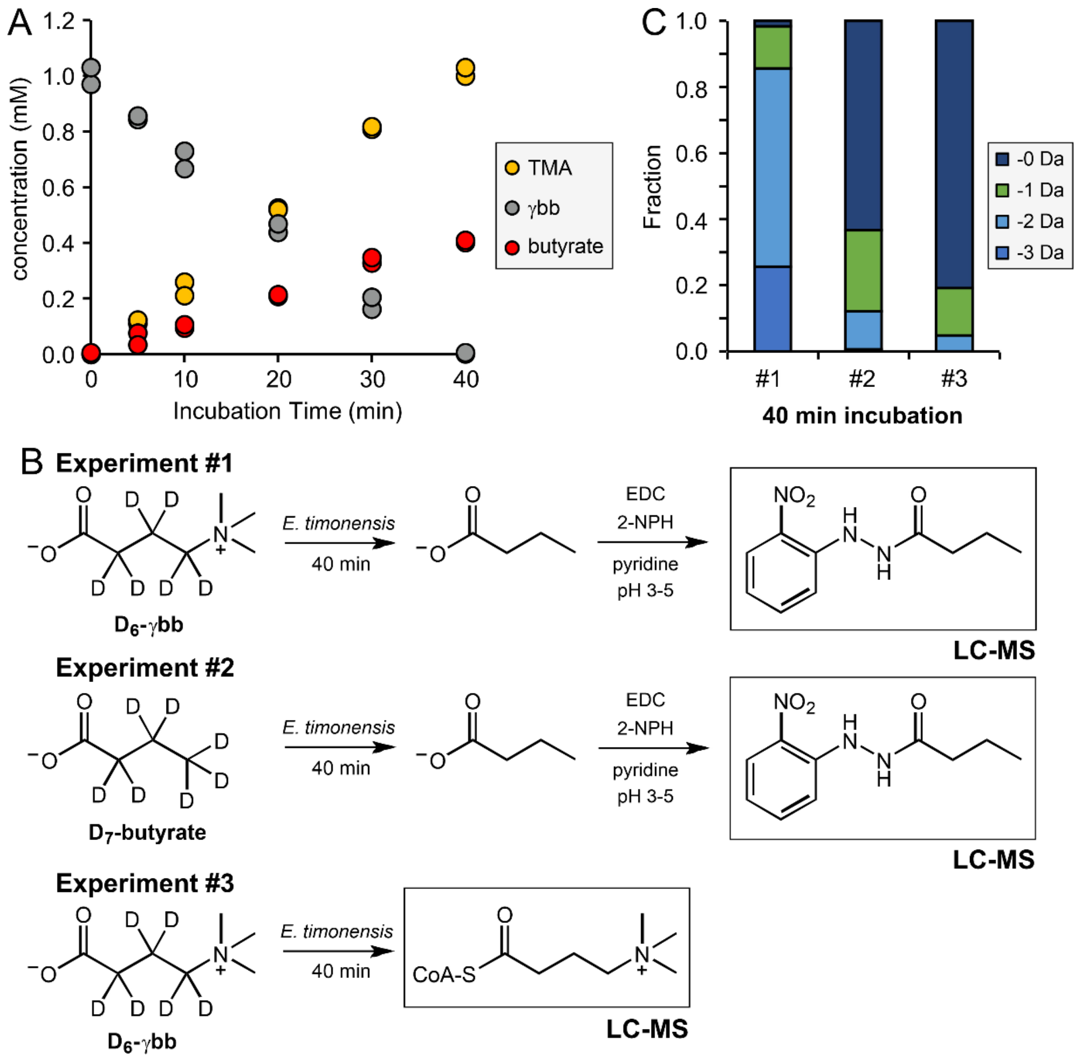

Figure S9. (**A**) Butyrate production during an incubation of 1 mM γbb in a resting suspension of *E. timonesis* SN18 cells grown in the presence of γbb. (**B**) Experimental conditions for results shown in panel C. Experiment #1 represents the mass spectrum of derivatized butyrate from a resting cell suspension of γbb-induced *E. timonesis* SN18 incubated with 1 mM D_6-γbb for 40 min. Experiment #2 represents the mass spectrum of derivatized butyrate from a resting cell suspension of γbb-induced *E. timonesis* SN18 incubated with 1 mM D_7-butyrate for 40 min. Experiment #3 represents the mass spectrum of γbb-CoA from a resting cell suspension of γbb-induced *E. timonesis* SN18 incubated with 1 mM D_6-γbb for 40 min. (**C**) Isotopolog distribution of metabolite products detected by LC–MS from the three experiments described in panel B.

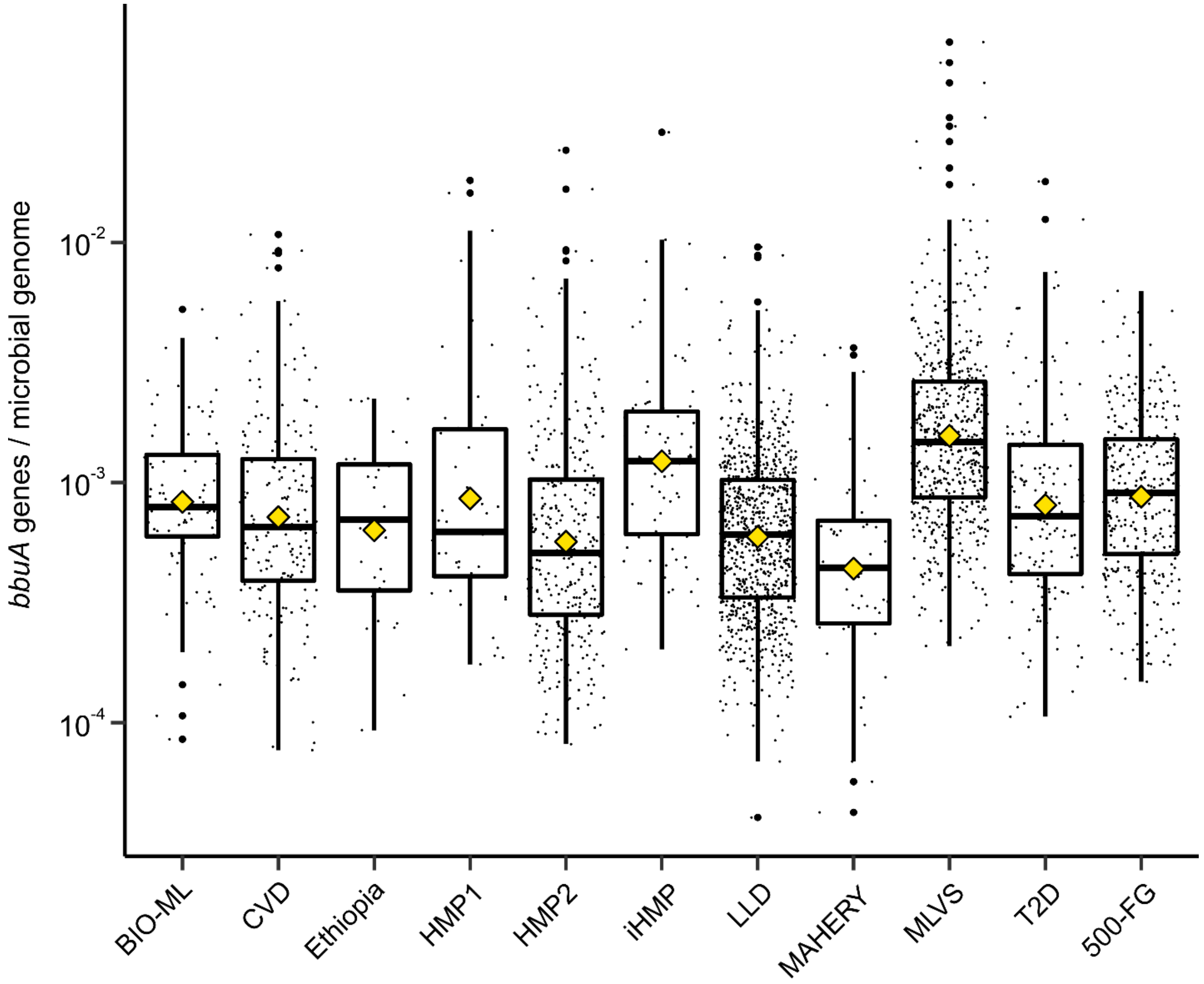

Figure S10. Abundance of the *bbuA* gene in stool metagenomes collected from various human studies. Mean values are represented by yellow diamonds.

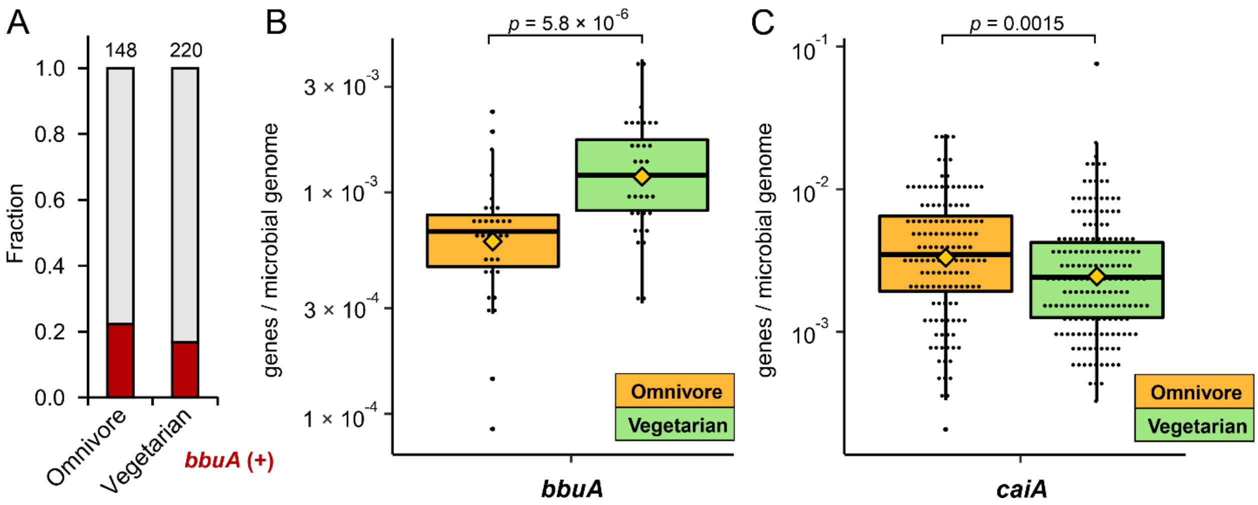

Figure S11. Correlation between γbb metabolism and diet. (**A**) Fraction of samples positive and negative for the *bbuA* gene in stool metagenomes of self-reported omnivores and vegetarians from the BIO-ML study. Comparison of (**B**) *bbuA* or (**C**) *caiA* gene abundance in stool metagenomes of self-reported omnivores and vegetarians from the BIO-ML study. Mean values are represented by yellow diamonds and *p*-values were determined using the Mann-Whitney *U*-test.

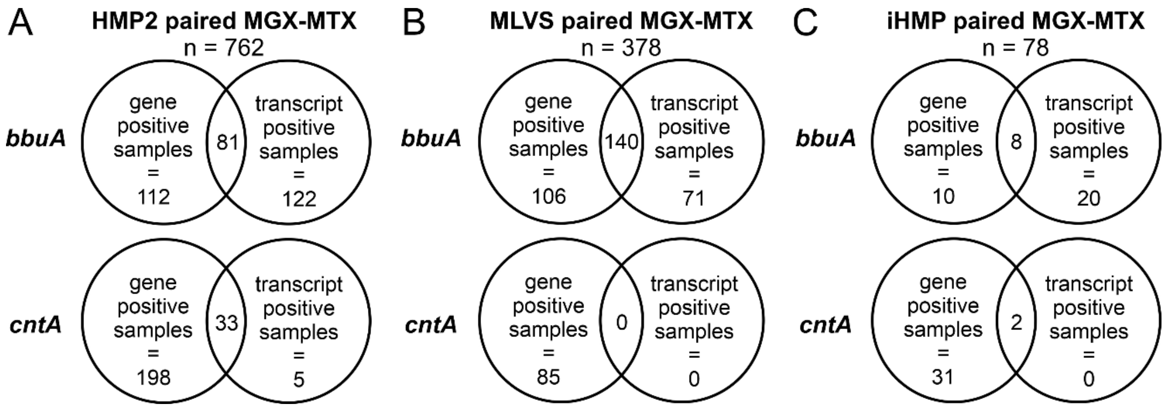

Figure S12. Overlap of stool samples from the (**A**) HMP2, (**B**) MLVS, and (**C**) iHMP cohorts that are positive for the *bbuA* or *cntA* gene and/or transcripts.

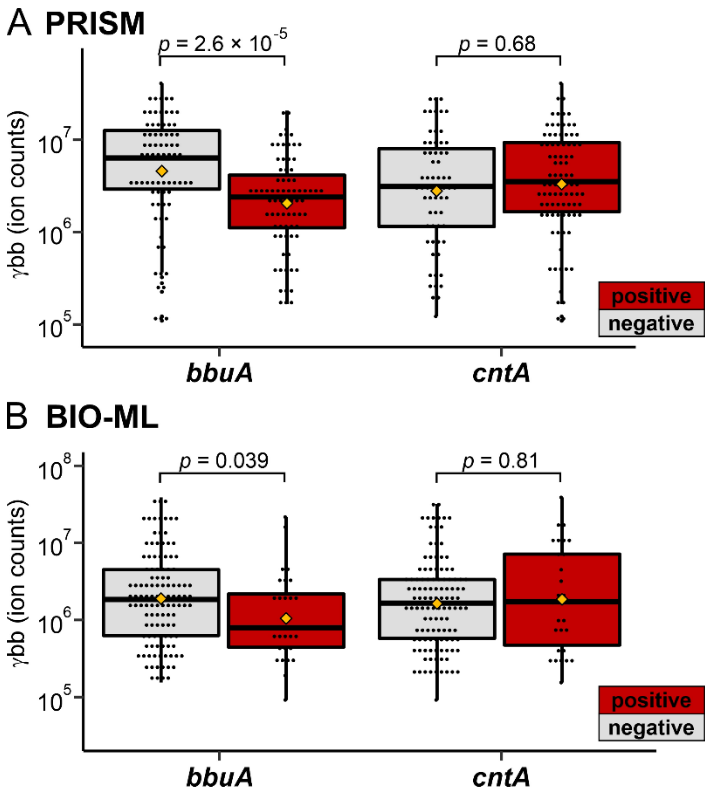

Figure S13. Correlations between the presence of *bbuA* or *cntA* in metagenomes and γbb metabolite levels in stool samples from the (**A**) PRISM and (**B**) BIO-ML cohorts. Mean values are represented by yellow diamonds and *p*-values were determined using the Mann-Whitney *U*-test.

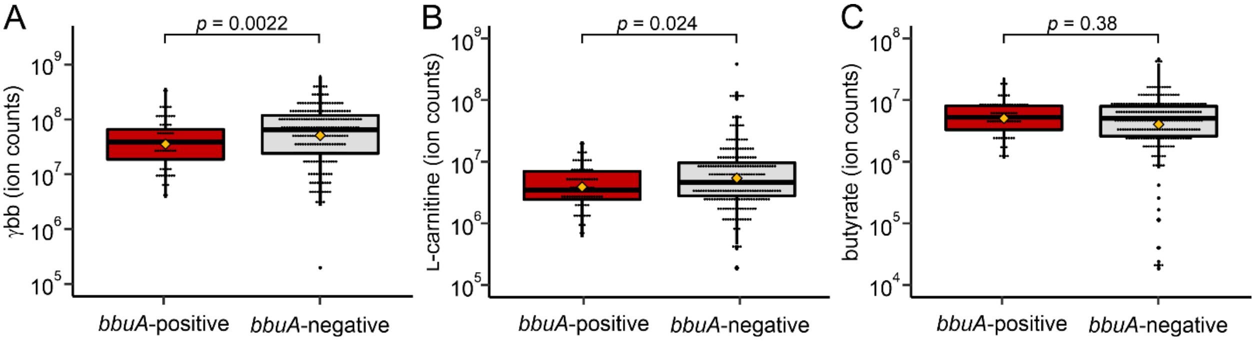

Figure S14. Correlations between the presence of *bbuA* in metatranscriptomes and levels of (**A**) γbb, (**B**) L-carnitine and (**C**) butyrate in stool metabolomes from the HMP2 projects. Mean values are represented by yellow diamonds and *p*-values were determined using the Mann-Whitney *U*-test.

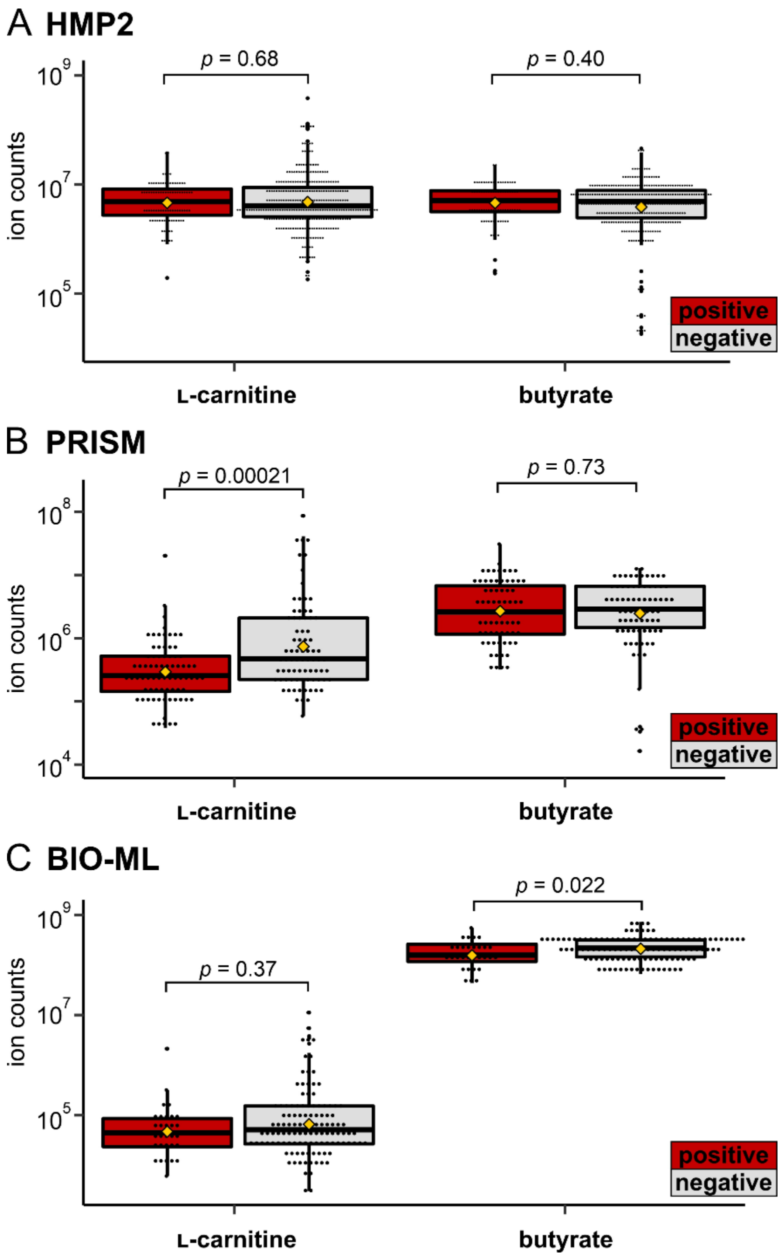

Figure S15. Correlations between the presence of *bbuA* in metagenomes and L-carnitine (orange) or butyrate (green) metabolite levels in stool samples from the (**A**) HMP2, (**B**) PRISM, and (**C**) BIO-ML projects. Mean values are represented by yellow diamonds and *p*-values were determined using the Mann-Whitney *U*-test.

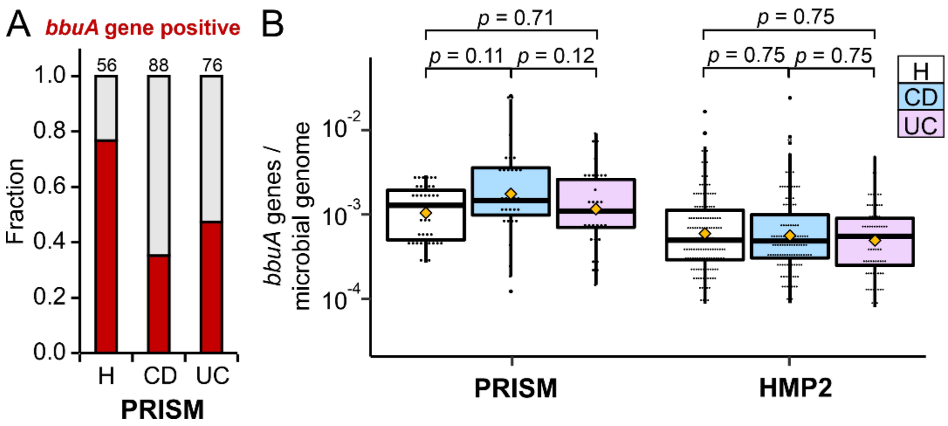

Figure S16. (**A**) Proportion of samples positive or negative for the *bbuA* gene in the PRISM Crohn's disease (CD) and ulcerative colitis (UC) cohorts compared to healthy (H) controls. (**B**) Abundance of the *bbuA* gene in IBD cohorts compared to healthy controls in the PRISM and HMP2 studies. Mean values are represented by yellow diamonds and *p*-values were determined using the Mann-Whitney *U*-test.

Dataset S1 (separate file). *E. timonensis* RNA-sequencing data statistics and gene expression (RPKM) data.

Dataset S2 (separate file). Homologs of enzymes responsible for crotonyl-CoA metabolism in *E. timonensis* and corresponding gene expression data from RNA-seq.

Dataset S3 (separate file). List of isolate genome collections and MAGs analyzed for the present of the *bbu* genes. MAGs containing *bbu* gene clusters are provided, including information about study and sample of origin, completeness, contamination, and taxonomy classification.

Dataset S4 (separate file). List of human studies with metagenomics datasets used for bioinformatic analyses.

Dataset S5 (separate file). Primer sequences and accession codes of gene sequences used in molecular cloning.

Dataset S6 (separate file). BbuA protein sequences used for bioinformatic analyses.
